# Dynamics of male canine germ cell development

**DOI:** 10.1371/journal.pone.0193026

**Published:** 2018-02-28

**Authors:** Aline F. de Souza, Naira C. Godoy Pieri, Kelly C. S. Roballo, Fabiana F. Bressan, Juliana B. Casals, Carlos E. Ambrósio, Felipe Perecin, Daniele S. Martins

**Affiliations:** 1 Department of Surgery, Faculty of Veterinary Medicine and Animal Sciences, University of São Paulo, São Paulo, SP, Brazil; 2 Department of Veterinary Medicine, Faculty of Animal Sciences and Food Engineering, University of São Paulo, Pirassununga, SP, Brazil; 3 Department of Reproduction, Faculty of Veterinary Medicine, University of São Paulo, São Paulo, SP, Brazil; Seoul National University, REPUBLIC OF KOREA

## Abstract

Primordial germ cells (PGCs) are precursors of gametes that can generate new individuals throughout life in both males and females. Additionally, PGCs have been shown to differentiate into embryonic germ cells (EGCs) after *in vitro* culture. Most studies investigating germinative cells have been performed in rodents and humans but not dogs (*Canis lupus familiaris*). Here, we elucidated the dynamics of the expression of pluripotent (*POU5F1* and *NANOG*), germline (*DDX4*, *DAZL* and *DPPA3*), and epigenetic (5mC, 5hmC, H3K27me3 and H3K9me2) markers that are important for the development of male canine germ cells during the early (22–30 days post-fertilization (dpf)), middle (35–40 dpf) and late (45–50 dpf) gestational periods. We performed sex genotype characterization, immunofluorescence, immunohistochemistry, and quantitative reverse transcriptase polymerase chain reaction (RT-qPCR) analyses. Furthermore, in a preliminary study, we evaluated the capacity of canine embryo PGCs (30 dpf) to differentiate into EGCs. To confirm the canine EGCs phenotype, we performed alkaline phosphatase detection, immunohistochemistry, electron and transmission scanning microscopy and RT-qPCR analyses. The PGCs were positive for *POU5F1* and H3K27me3 during all assessed developmental periods, including all periods between the gonadal tissue stage and foetal testes development. The number of *NANOG*, DDX4, DAZL, DPPA3 and 5mC-positive cells increased along with the developing cords from 35–50 dpf. Moreover, our results demonstrate the feasibility of inducing canine PGCs into putative EGCs that present pluripotent markers, such as POU5F1 and the *NANOG* gene, and exhibit reduced expression of germinative genes and increased expression of H3K27me3. This study provides new insight into male germ cell development mechanisms in dogs.

## Introduction

During embryonic development, extensive reprogramming occurs via gene regulation, resulting in changes in cell dynamics and the generation of new cells, such as primordial germ cells (PGCs) [1]. Because PGCs are precursors of gametes that are capable of generating new individuals and transmit genetic material to future generations [2]. Studies investigating PGCs have revealed interesting findings regarding their migration route, *in vitro* cultivation and differentiation [3,4]. Furthermore, fertility in humans and other species relies on the successful development of PGCs [5].

PGCs originate from a small population of cells in the extra-embryonic mesoderm in the posterior region in E7.0–7.25 mice [6]. In humans, PGCs development begins during the third week of gestation in the posterior region of the yolk sac at the allantois base [7]. As the mammalian embryo develops, the PGCs migrate into the embryo towards the somatic cells of the developing gonadal ridge, where they are reorganized to form future gametes [8,9].

The beginning of the sexual differentiation process is characterized by the formation of seminiferous cords in males and ovarian cords in females [10]. In particular, the morphological process of female gonad development occurs slowly as the cortical cords extend towards the superficial epithelium of the gonad incorporating the PGCs that are around the follicular cells, transforming the PGCs into oocytes [11].

During the morphological transformation of the male gonads develop into rete testes, the male PGCs also specialize and gradually form a distinct population of gonocytes and pre-spermatogonial cells (or germ cells) [12]. Pre-spermatogonial cells continue their mitotic proliferation and enter meiosis only at puberty, whereas the oogonia enter the meiotic prophase and differentiate into primary oocytes. Male germ cells advantageously continuously express the pluripotent markers *POU5F1* (or *OCT4*) and *NANOG* and other germline markers, such as *DDX4* (*VASA*) and *DAZL* [13–15]. Thus, male PGCs are useful for studying pluripotency acquisition and maintenance. Furthermore, male PGCs have been used in gamete production *in vitro* and can be used as an alternative to reproduction biotechnology strategies, such as *in vitro* fertilization (IVF) and transgenic animal generation [16].

Several studies have reported that inducing PGCs into embryonic germ cells (EGCs) is possible [17–20]. Such reprogramming is extremely desirable because EGCs have properties that are similar to those of embryonic stem cells (ESCs), thus enabling an understanding of the acquisition process of epigenetic modifications and providing support for reproductive medicine studies and therapies [4]. Most studies investigating PGCs and EGCs were conducted in mice [21,22], and only a few studies have been conducted in small, domestic animals, such as canines (dogs, *Canis lupus familiaris*). Gier and Marion demonstrated that canine PGCs migrate through the mesenchyme between the coelomic epithelium at 20–21 days of gestation [23]. Recently, the progression of distinct PGCs populations during gonadal morphological change has been observed in canine foetal testes [24]. Interestingly, *in vitro* EGCs derivation in canines has never been reported.

Dog models are considered ideal animal models of disorders or diseases that affect humans because canines are afflicted by innumerable genetic disorders and chronic degenerative diseases that are similar to those experienced by humans [25,26]. In addition, animal models, such as dogs, can be used in the fields of stem cell research and therapy [27–29]. Furthermore, at least half of the more than 400 hereditary canine diseases are shared by humans [30–31]; thus, dogs represent an acceptable translational research model. Due to the similarities between canines and humans [32] and the availability of canine PGCs material, it is both important and desirable to explore this model. We applied a global approach to male canine PGCs and identified a profile with different pluripotent (*POU5F1* and *NANOG*), germinative (*DDX4*, *DAZL* and *DPPA3*), and epigenetic markers (5mC, 5hmC, H3K27me3 and H3K9me2) throughout the early (22–30 days post-fertilization (dpf)), middle (35–40 dpf) and late (45–50 dpf) stages of pregnancy, thereby revealing the developmental progression of PGC populations during male foetal development. We also demonstrated the feasibility of culturing canine PGCs (30 days dpf) *in vitro*, which allowed us to observe the microenvironment and determine that PGCs could be differentiated into putative canine EGCs.

## Materials and methods

### Canine germ cell identification and collection

All study procedures were performed in accordance with the Guide for the Care and Use of Laboratory Animals of the National Institutes of Health, and all procedures involving animals were conducted in accordance with the Committee of Ethics of the Veterinary Medicine School at the University of São Paulo, Brazil (protocol number 13.1.1729.74.2). The canine embryo and foetal male gonad samples were obtained from healthy pregnant female mongrel dogs between one to five years of age. The pregnant dogs (n = 20) obtained from a dog population control campaign in São Paulo and Pirassununga-São Paulo, Brazil, underwent ovary-salpingo-hysterectomy surgery. Before performing any surgical procedure, the animals were food restricted for 12 hours and water restricted for 8 hours. Anaesthesia was performed as previously described [32], with some modifications: muscle relaxant (0.05 mg/kg acepromazine + 2 mg/kg meperidine–intramuscular– 1X), anaesthetic (5 mg/kg propofol–intravenous– 1X), and inhaled anaesthesia (1.5–2.5% isoflurane–continued throughout the surgery). The post-surgical treatment was as follows: antibacterial therapy (20–30 mg/kg cephalexin–oral–once every 12 hours), analgesics (1–4 mg/kg tramadol–intramuscular–once every 12 hours), anti-inflammatory (0.1–0.2 mg/kg meloxicam–oral– 1X) and local care (rifamycin). The dogs were maintained alive after the procedure and all animals were placed for adoption.

The uteri used after the ovarian hysterectomies were washed with phosphate buffer solution (PBS) to remove mucus and cell debris. After this procedure, the uteri were dissected and incised, and the extra-embryonic membranes were bathed to expose and remove the embryos and foetuses. The gestational age of the collected embryos and foetuses was determined based on the morphogenesis development and the crown-rump (CR) measurements [33]. The embryos were collected between 22 and 30 days of gestation (early), which corresponds to the gestational stage prior to sexual differentiation. The foetuses were collected between 35 and 40 days (middle), which corresponds to the beginning of sexual differentiation, and between 45 and 50 days (late), which corresponds to the period during which the gonads develop into testes precursors.

### Sex genotype assay

The sex genotyping of the collected canine tissues was performed as previously described [34]. Using early gestation samples, the sex genotyping was achieved by performing PCR with primers specific to the *SRY* gene (forward sequence 5′-AAG GCC ACG GCA CAG AAA AGT CAC 3’, reverse sequence 5′-AAG AAG CGT CAG CGG ACA TCT GTG) [35,36]. The PCR conditions consisted of an initial denaturation step at 93°C for 3 minutes followed by 35 cycles of denaturation at 93°C for 40 seconds, annealing from 54 to 65°C for 40 seconds, elongation at 72°C for 1 minute and a final elongation step at 72°C for 10 minutes. The PCR products were visualized on a 2% agarose gel. The sex of the canine foetal gonads in the middle and late pregnancy stages was determined by a visual *in situ* inspection of the gonads.

### Detection and analysis of proteins related to pluripotency and germinative and epigenetic reprogramming during male canine PGC development

The detection of DDX4, DAZL, DPPA3, POU51F (OCT4), SOX2, 5mC, 5hmC, H3K27me3 and H3K9me2 in the male canine PGCs was achieved by performing immunofluorescence and immunohistochemistry during the gestational period to elucidate the dynamic profiles of pluripotency- and epigenetic-related marker expression.

In the immunofluorescence assay, the embryos in the early (n = 7), middle (n = 3) and late (n = 4) stages were evaluated in biological triplicate. The samples were fixed in a 4% paraformaldehyde solution (PFA) for 24 hours, dehydrated in 70–100% (v/v) ethanol, embedded in paraffin, sliced into 5-μm-thick sections using a microtome, and mounted on glass slides. The mounted canine testis tissue was rehydrated with xylene. The nucleus and cytoplasm in the rehydrated samples were stained using haematoxylin and eosin (H&E).

Immunofluorescence staining was performed as previously described [10] using 0.01 M citrate buffer at pH 6.0 for the antigen retrieval. Prior to applying the primary antibodies, the slides were blocked with 1% bovine serum albumin (BSA, Sigma-Aldrich Corp., St. Louis, MO, USA) and 0.1% PBS Tween-20 (PBS-T) for one hour at room temperature. The primary antibodies included polyclonal anti-rabbit IgG anti-DDX4 (VASA) (1:500, ab13840, Abcam, Cambridge, England), polyclonal anti-rabbit IgG anti-DAZL (1:200, ab34139, Abcam, Cambridge, England), polyclonal anti-rabbit IgG anti-DPPA3 (STELLA) (1:500, sc67249, Santa Cruz Biotechnology, CA, USA), polyclonal goat IgG anti-POU51F (OCT4) (1:100, sc-8629, Santa Cruz Biotechnology, CA, USA), polyclonal anti-rabbit IgG anti-SOX2 (1:100, ab97959, Abcam, Cambridge, England), monoclonal mouse IgG anti-5mC (1:500, ab10805, Abcam, Cambridge, England), polyclonal anti-rabbit IgG anti-5hmC (1:100, ap9160a, Abgent, San Diego, CA, USA), polyclonal anti-rabbit IgG anti-H3K27me3 (1:500,7449, Millipore, Temecula, CA, USA), and polyclonal anti-rabbit IgG anti-H3K9me2 (1:500, 7441, Millipore, Temecula, CA, USA). One embryo (15 dpf) was used as a positive control for the DPPA3 antibody, histone repressive marker antibodies, such as H3K27me3 and H3K9me2, and antibodies indicative of DNA methylation (5mC) and DNA hydroxymethylation (5hmC) (S1 Fig). Pre-pubertal and adult canine testes were used for the DDX4 antibody positive control (S2 Fig). The DAZL antibody has been previously used and validated [37].

The secondary antibodies included Alexa Fluor 488 donkey anti-rabbit (1:500, A21206, Life Technologies), Alexa Fluor 594 donkey anti-goat (1:500, A11058, Life Technologies), and Alexa Fluor 594 donkey anti-mouse (1:500, A-21203, Life Technologies) and were applied for one hour at room temperature. The negative controls were obtained by omitting the primary antibodies. The nuclei were stained with a Hoechst dye (trihydrochloride, trihydrate, cat# 33342, Invitrogen, Carlsbad, CA, USA), mounted with Prolong Gold antifade (cat# P36930, Life technology; Carlsbad, CA, USA), and stored at 4°C. All data were acquired under a fluorescence light microscope (FLM) (Nikon Eclipse 80i) at 60X and 100X magnification. ImageJ (National Institutes of Health, USA) was used for the quantification analyses. The images were first converted to 8-bit greyscale images, and colour inversion was applied to selected images that required increased sensitivity. The images were then quantified using “analyses particles”, which allowed for single-cell counting.

To detect 5mC using immunohistochemistry, the samples obtained during the early (n = 7), middle (n = 3) and late (n = 4) stages were fixed in 4% PFA buffer for 24 hours, dehydrated and embedded in paraffin for the histological and immunohistochemistry analyses. The antigen retrieval was achieved by microwaving the slides using 0.01 M citrate buffer at pH 6.0 for 12 minutes at 92°C. The slides were blocked with 0.3% hydrogen peroxidase diluted in a tris buffer solution (TBS– 2.0 mM Trizma base and 1.36 mM sodium chloride, pH 7.5) for 20 minutes and then incubated with the monoclonal mouse IgG anti-5mC primary antibody (1:500, ab10805, Abcam, Cambridge, England) overnight at 4°C. The primary antibody was omitted for the negative control. The reaction was revealed using the Dako Advance HRP commercial kit (cat# K4069 HRP, Dako, USA) and 3,3′-diaminobenzidine (DAB) (DAB Peroxidase Substrate Kit, Vector Laboratories, Burlingame, CA, USA, #SK-4100). The slides were analysed under a Zeiss KS400 light microscope (Zeiss, Göttingen, Germany), and the images were captured at 10X and 40X magnification using AxioVision 4.6 software.

### Analysis of pluripotent and germline gene expression by RT-qPCR

Total RNA was isolated from three animals at each stage (early, middle and late) using the TRIzol reagent (Life Technologies) according to the manufacturer’s recommended protocol. The RNA was quantified according to the 260/280 ratio using a spectrophotometer (DS-11-B, DeNovix). cDNA synthesis was performed with a total of 1000 ng/μl per sample using a High-Capacity Reverse Transcription Kit (Applied Biosystems, CA, USA) according to the manufacturer’s recommended protocol. The primer sequences (Table 1) used for the pluripotency (*POU5F1* and *NANOG*) and house-keeping glyceraldehyde 3-phosphate dehydrogenase (*GADPH*) analyses have been previously described [38]. Other gene-specific primer were designed using Primer 3 [39] (S3 Fig). The PCR amplification of the selected pluripotent and germinative genes (Table 1) was performed using an ABI-7500 instrument with SYBR Green Master Mix (Applied Biosystems).

**Table 1:** Sequences of the primers used for RT-qPCR.

Genes (Accession n°)	Forward primer sequence	Reverse primer sequence	Annealing (°C)
*GADPH* (NM_001003142.2)	CTTCACCACCATGGAGAAGC	CAGCTCAGGGATGACCTTGC	60
*POU5F1* (XM_538830.3)	ATATGTGTAAGCTGCGGCCC	CAATGTGGCTGATCTGCTGC	62
*NANOG* (XM_022411387.1)	TCTGCCACCACGGAATATGC	TCTGACTGTTCCAGGAGTGG	60
*DDX4* (XM_005617393.2)	CATACCACCTCCTCCACCTG	TGTCTGACAGAGATTAGCTTCCTC	59.60
*DAZL* (XM_005634380.2)	ACTGGTGTGTCCAAAGGCTAT	GGACGAGGCTGCACATGATA	59.90
*DPPA3* (XM_022411213.1)	AACTCCCTTCCCCTCTACCA	CGCTGGTACTGAATCAATCG	59.60

RT-qPCR was performed as follows: 95°C for 15 minutes and 45 cycles at 95°C for 15 seconds, 60°C for 5 seconds, 72°C for 30 seconds and finally 72°C for 2 minutes. A melting curve analysis was performed to verify the amplification of the specific products, and all reactions were performed in triplicate. The transcripts levels were determined by RT-qPCR and analysed using LinReg PCR software (Version 2015.0). The cycle threshold (Ct) values of the target genes were normalized to the Ct value of GAPDH, and then, the fold changes were calculated using the Pfaffl equation [40]. The statistical analyses included an analysis of variance (ANOVA, p<0.1) followed by Tukey’s test to determine the differences in gene expression and the group means (p<0.1) using R software [41]. A Pearson correlational analysis (p<0.05) was performed to verify the gene expression correlations among the different stages.

A standard nucleotide–nucleotide BLAST (blastn) search was conducted to verify the similarity between the sequence obtained in this study and other sequences available in the GenBank database [42]. A phylogenetic analysis was performed using maximum likelihood (ML) methods and a Kimura 2-parameter (K2P) evolution sequence model with the MEGA 6.0 programme [43]. A bootstrap analysis (1000 replicates) was performed to assess the relative robustness of the tree branches (S1 Table) (S3 and S4 Figs).

### Isolation of canine putative EGCs

The dissection of the canine foetal gonad samples at the middle pregnancy stage (30 days) (n = 15) was performed using stereomicroscopy under sterile conditions. The gonadal ridge was enzymatically dissociated using three different protocols. The first protocol consisted of mechanical dissociation followed by enzymatic dissociation using 0.5% trypsin and 1 mg/ml collagenase IV at 37°C for 10–15 minutes. The second protocol consisted of mechanical dissociation followed by enzymatic dissociation using 0.5% trypsin and 0.5 mM EDTA at 37°C for 10–15 minutes. The third protocol consisted of mechanical dissociation using a scalpel blade followed by a PBS wash and incubation with 1 mg/ml dispase (Gibco), 0.3 mg/ml hyaluronidase (Sigma) and 0.5 mM EDTA diluted in PBS at 37°C for five minutes. After the gonads were completely dissociated, the cells were washed with PBS and plated in 24-well tissue culture plates (1x10^4^ cells per well) previously coated with gelatine and canine embryonic fibroblasts (CEFs) mitotically inactivated by mitomycin C (Sigma). The cells were cultured in Knockout Dulbecco’s Modified Eagle Medium (KO/DMEM) (Gibco) supplemented with 10% Knockout Serum Replacement (KSR, Gibco), 0.1 mM nonessential amino acids (Gibco BRL), 0.1 mM beta-mercaptoethanol (Gibco BRL), 2 mM L-glutamine (Gibco BRL), 50 μg/ml penicillin/streptomycin, and 1 mM sodium pyruvate (Gibco BRL).

To promote development and proliferation, the EGCs were cultured with the following supplements: 4 ng/ml human basic fibroblast growth factor (bFGF) (Cell Science; Norwood, MA, USA), 1000 U/ml human leukaemia factor inhibitor (LIF) (Chemicon International), and 10 μM forskolin (Sigma). On the 3^rd^, 10^th^, 15^th^ and 20^th^ days of culture, the cells were manually disaggregated, and fresh CEFs and medium were supplied. All cultures were maintained at 5% CO_2_ and 38.5°C.

### EGCs characterization

The same proteins analysed in the PGCs were analysed using immunocytochemistry, alkaline phosphatase detection and RT-qPCR to confirm the canine EGCs lineages, pluripotency and epigenetic status.

In the immunocytochemical analysis, each protein was measured in triplicate. The EGCs colonies (n = 20) were fixed in 4% PFA for 12 minutes, and the cells were permeabilized with PBS and 0.1% Triton X-100 (TBST, Sigma) for 20 minutes. The cells were blocked with BSA (Sigma) for one hour. The same primary and secondary antibodies used for the immunofluorescence were used for the immunocytochemistry analysis, and one additional primary antibody (THY-1, 1:100, sc-6071, Santa Cruz Biotechnology, CA, USA) was used. The primary antibodies were incubated for one hour at room temperature and then washed three times with PBS. Subsequently, the secondary antibodies were incubated for one hour at room temperature. The negative controls were obtained by omitting the primary antibodies. The cells were counterstained with a 1:1000 dilution of Hoechst dye (trihydrochloride, trihydrate, cat# 33342, Invitrogen, Carlsbad, CA, USA) and mounted with Prolong Gold antifade (cat# P36930, Life technology; Carlsbad, CA, USA). All data were acquired under a Leica SP5 confocal microscope (Leica TCS SP5, Leica Microsystem) at 20x and 40X magnification.

For the alkaline phosphatase (AP) detection, the cells were washed three times with PBS and analysed using a commercial kit (Leukocyte Alkaline Phosphatase Kit, Sigma, cat. # 86R) according to the manufacturer's recommended protocol.

The RT-qPCR analysis of the *DDX4* and *NANOG* genes was performed as follows: 95°C for 15 minutes and 45 cycles at 95°C for 15 seconds, 60°C for 5 seconds, 72°C for 30 seconds and finally 72°C for 2 minutes. A melting curve analysis was performed to verify the amplification of the specific products, and all reactions were performed in triplicate. The analysis was performed using the same protocol as for the analysis of the PGCs. The statistical analyses included *t-tests* (p<0.05) performed using R software [41].

### Scanning and transmission electron microscopy analyses of putative EGCs

A previously established scanning electron microscopy (SEM) assay was performed with some modifications [44]. In brief, the EGC colonies were fixed with 2.5% glutaraldehyde in culture plates for two hours at 4°C and then dehydrated in a graded acetone series. The samples were then dried in an oven for two hours, metallized in a gold bath with a metallizer (Emitech K 550) and examined under a scanning electron microscope (Leo—435 VP Zeiss). The transmission electron microscopy (TEM) protocol has been previously established [45]. Briefly, the EGCs were fixed in 2.5% glutaraldehyde diluted in PBS (pH 7.4–0.1 M) for 24 hours. The fragments and cells were then washed in PBS, post-fixated in osmium tetroxide (4% w/w solution in water, Polysciences, Inc., USA) for one hour, and washed with PBS again. The fragments and cells were then dehydrated in increasing concentrations of ethanol (70–100%) using propylene oxide (EM Grade, Polysciences) as the final dehydration reagent. The samples were incubated in a 1:1 mixture of propylene oxide and Spurr’s resin (Spurr’s kit, Electron Microscopy Sciences) at 60°C for 12–16 hours and then 100% Spurr’s resin for an additional five hours with constant agitation. The blocks were cut using an ultra-microtome (Leica VR model Ultracut UCT). Semi-thin 1-mm sections were obtained and stained with a hot aqueous solution of 1% sodium borate in distilled water containing 0.25% toluidine blue for observation under a light microscope. Ultrathin cuts of approximately 60 nm in thickness were collected on copper screens and constricted by 2% uranyl acetate in distilled water for 10 minutes and 0.05% lead citrate in distilled water for 10 minutes. The subcellular observations and electromicrographs were performed under an electron microscope (Morgagni 268D, FEI Company, The Netherlands; Mega View III camera, Soft Imaging, Germany).

## Results

### Characterization of canine PGCs during development

We visualized the formation of the gonadal ridge in samples obtained during the early stage of gestation. The urogenital system extends caudally towards the upper region of the abdominal area around the caudal region of the hind legs. During this stage, we observed the mesonephros, which is located laterally to the urogenital ridge system, with prominent tubules. The tubules comprised epithelial cells, which formed the simple cubic epithelium and were wrapped around blood vessels. In the caudal region of this organ, we verified the presence of metanephric tissue, which is the primitive kidney. The primary or primitive gonads were located in the medial body region and were designated "undifferentiated" because we could not determine the sex of the embryo through morphological or histological analyses. Thus, the sex of the embryos was determined by performing genotyping assays. A 271-bp fragment produced from the PCR assay corresponded to the SRY gene, identifying the male gonads (S5 Fig).

**Fig 1:**
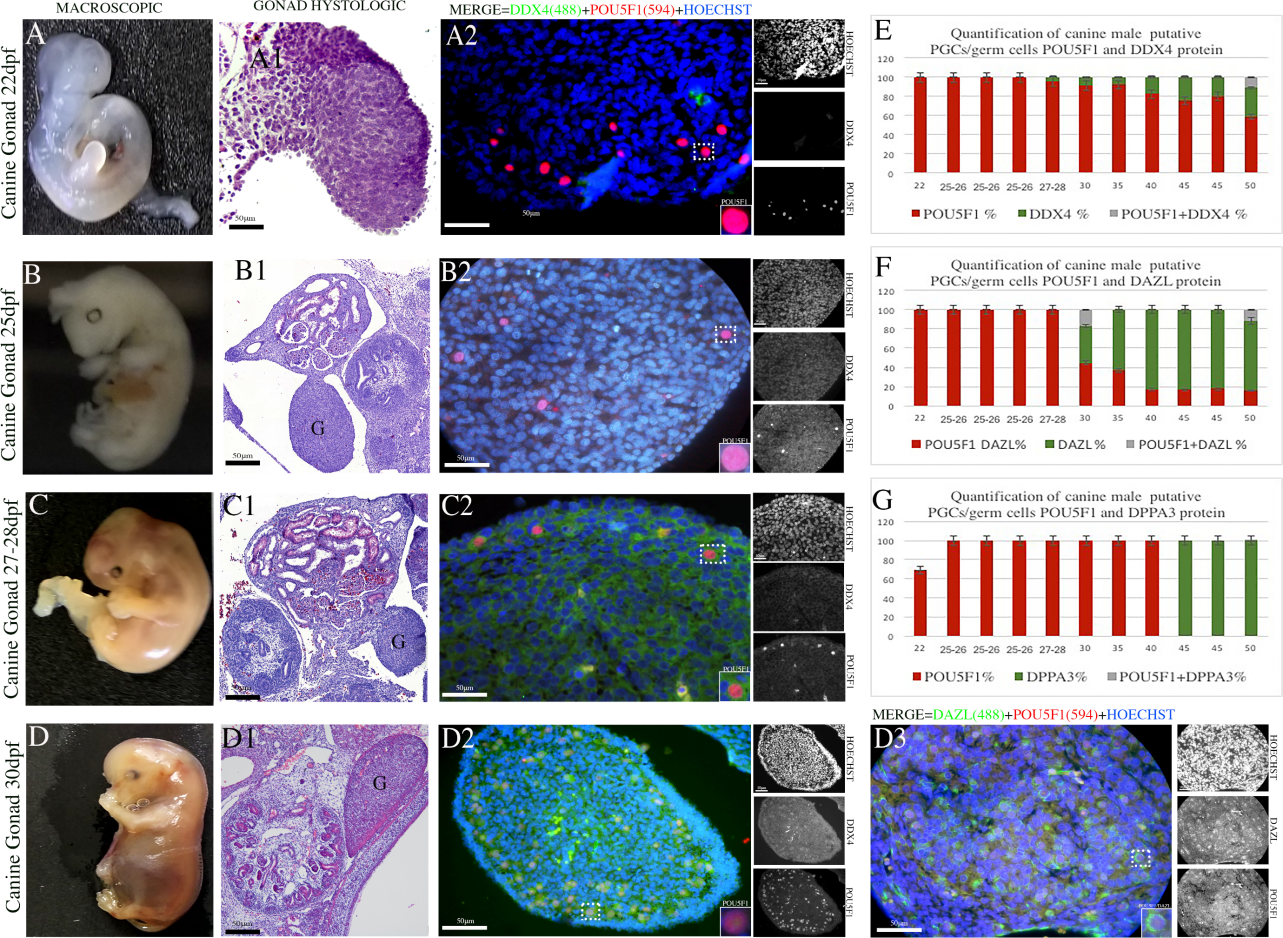
Dynamics of PGCs during the early development period of the male canine gonadal ridge. (A-D) Photomicrographs of sections of canine embryos at 22, 25, 27–28 and 30 days post-fertilization (dpf). (A1-D1) Histological section of male canine gonadal ridge during the early gestation period (22–30 dpf) (Scale bars are 50 μm). Sections of putative PGCs at 22, 25, 27–28 and 30 dpf immunoassayed for the early germ cell marker (nuclear) POU5F1 (red) and the late germ cell marker (cytoplasmic) DAZL (green) (Scale bars are 50 μm). (E) Percentage of quantified canine putative PGCs in canine gonads between 22 and 50 dpf detected using the POU5F1 and DDX4 antibodies. (F) Percentage of quantified canine putative PGCs in canine gonads between 22 and 50 dpf detected using the POU5F1 and DAZL antibodies. (G) Percentage of quantified canine putative PGCs in canine gonads between 22 and 50 dpf detected using the POU5F1 and DPPA3 antibodies.

The putative PGCs in the male gonads exhibited low POU5F1^+^ (red) expression as determined by the immunofluorescence staining (Fig 1), and certain positive cells were identified during the formation of the mesentery and dorsal aorta (S2 and S3 Tables). These positive cells were spherical or round in shape and exhibited a morphologically large nucleus that resembled that of germ cells. DPPA3- (green) and DDX4- (green) positive germ cells could not be detected in the samples obtained during the first stage of gestation. The specific germ cell marker DAZL was observed only in the gonadal ridges of the 30-day embryos. Cytoplasmic staining and the co-localization of the DAZL (green) and POU5F1^+^ (red) proteins were detected in the PGCs (Fig 1) (S3 Table), indicating that canine PGCs enter the cell maturation process, including gamete sexual differentiation, during this period.

**Fig 2:**
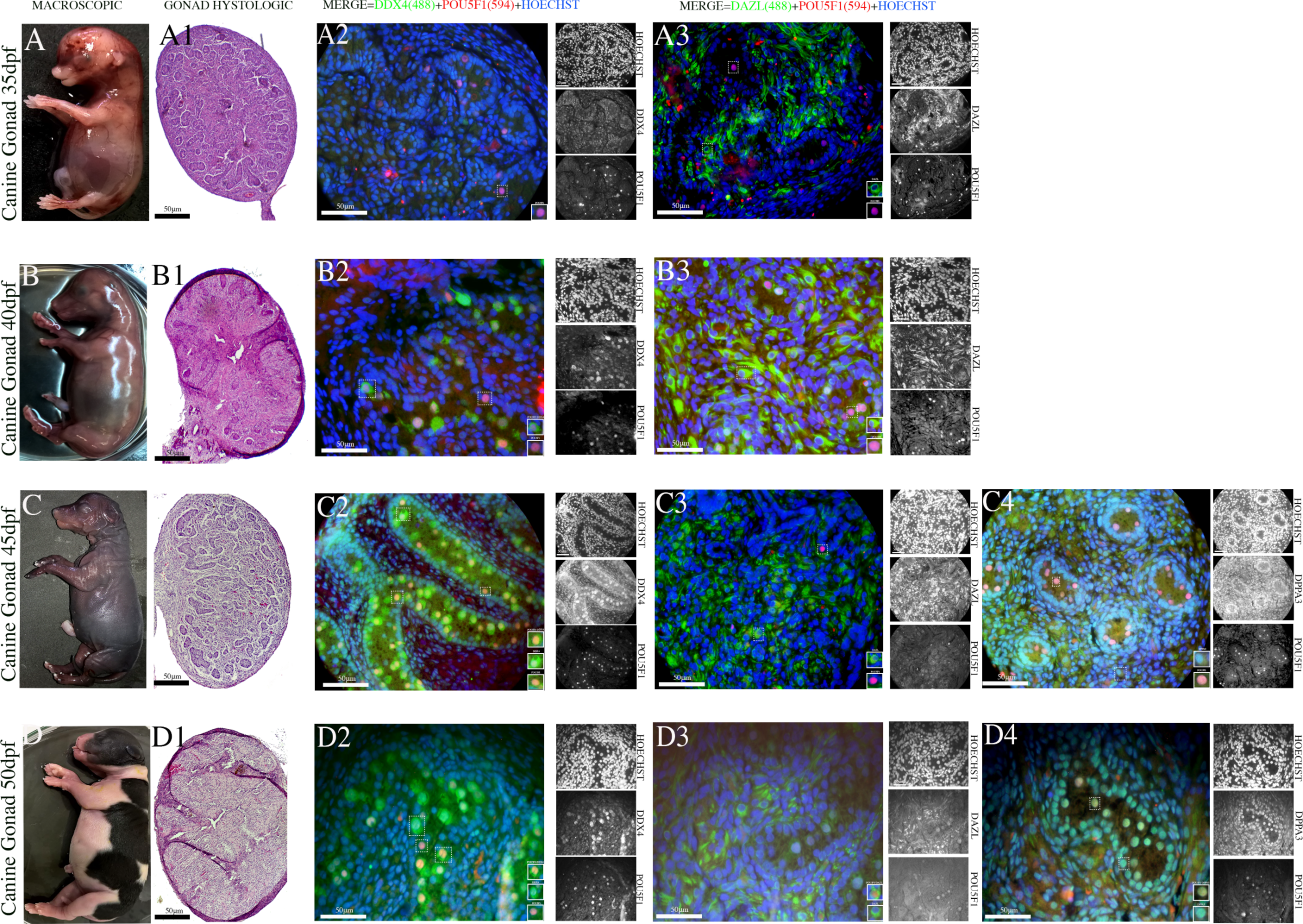
Dynamic expression of PGCs during the intermediate and late development periods of the male canine gonadal ridge. (A-D) Photomicrographs of sections of canine embryos at 35, 40, 45 and 50 dpf. (A1-D1) Histological section of male canine gonadal ridge during the early gestation period (35–45 dpf) (Scale bars are 50 μm). (A2-D2/A3-D3/C4-D4) Testes sections at 35, 40, 45 and 50 dpf immunoassayed for the early germ cell marker (nuclear) POU5F1 (red) and/or the late germ cell markers (cytoplasmic) DDX4, DAZL and (nuclear) DPPA3 (green) (Scale bars are 50 μm).

The middle period is marked by the initiation of sexual differentiation, during which the cells can be morphologically distinguished as males or females. The male gonads undergo major morphological changes, medullary cords are differentiated into seminiferous cords, and interstitial cells emerge at 35 days of gestation. During this stage of gestation, POU5F1 was detected in the nucleus of germinative cells within the seminiferous cords (Fig 2). While most germ cells were POU5F1^+^ (red) and DAZL^+^ (green) (Fig 2) (S3 Table), a few cells were DDX4^+^ (green) (S1 Table), and no cells were DPPA3^+^.

During the late period, gonads exhibit simple testes precursors, which are called foetal testes. The testicular cords vary in size, and certain pre-spermatogonial cells are present inside the testicular cords. In the foetal testes, we observed DDX4^+^ (green) cells, DAZL^+^ (green) cells and the co-localization of the nuclear markers with POU5F1 through single and double labelling (Fig 2) (S2 and S3 Tables). The nuclear protein DPPA3 (green) and/or co-localization of POU5F1 (yellow/orange) are emphasized. Additionally, DPPA3 was observed in the cords during the late stage, but its expression was also detected in the somatic cells surrounding the cords (Fig 2) (S3 Table).

### Gene expression analysis of gonads during male development by RT-qPCR

**Fig 3:**
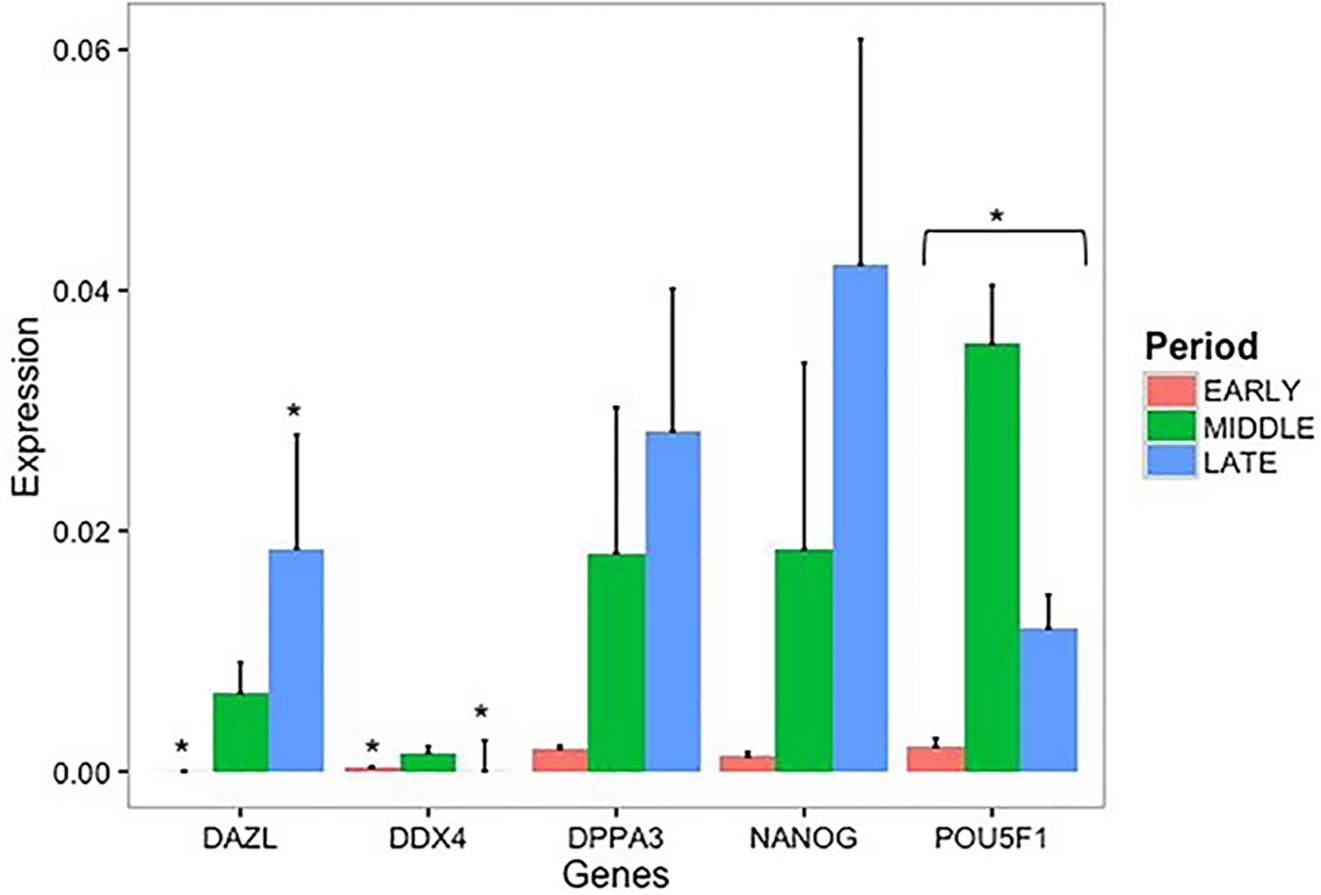
RT-qPCR analysis of several key pluripotent and germ cell-associated genes in PGCs among the periods. *POU5F1*, *NANOG*, *DDX4*, *DPPA3* and *DAZL* were normalized to the *GADPH* gene (N = 3 biological samples with technical triplicates for each period, p<0.1). Asterisks denote statistically significant differences among the periods.

The expression of important development-related genes varies according to the developmental stage and maturation of the PGCs in the gonads. Thus, we performed RT-qPCR analyses of the canine gonads during the early, intermediate/middle and late stages of canine development and detected the expression of almost all studied genes. An *ANOVA* revealed significant differences in the expression of the *POU5F1*, *DDX4* and *DAZL* genes, and *Tukey’s* test demonstrated significant differences in the expression of the *POU5F1* gene between the early stage and the middle and late stages. Furthermore, the expression of the *DDX4* and *DAZL* genes significantly differed between the early and late stages (Fig 3).

**Fig 4:**
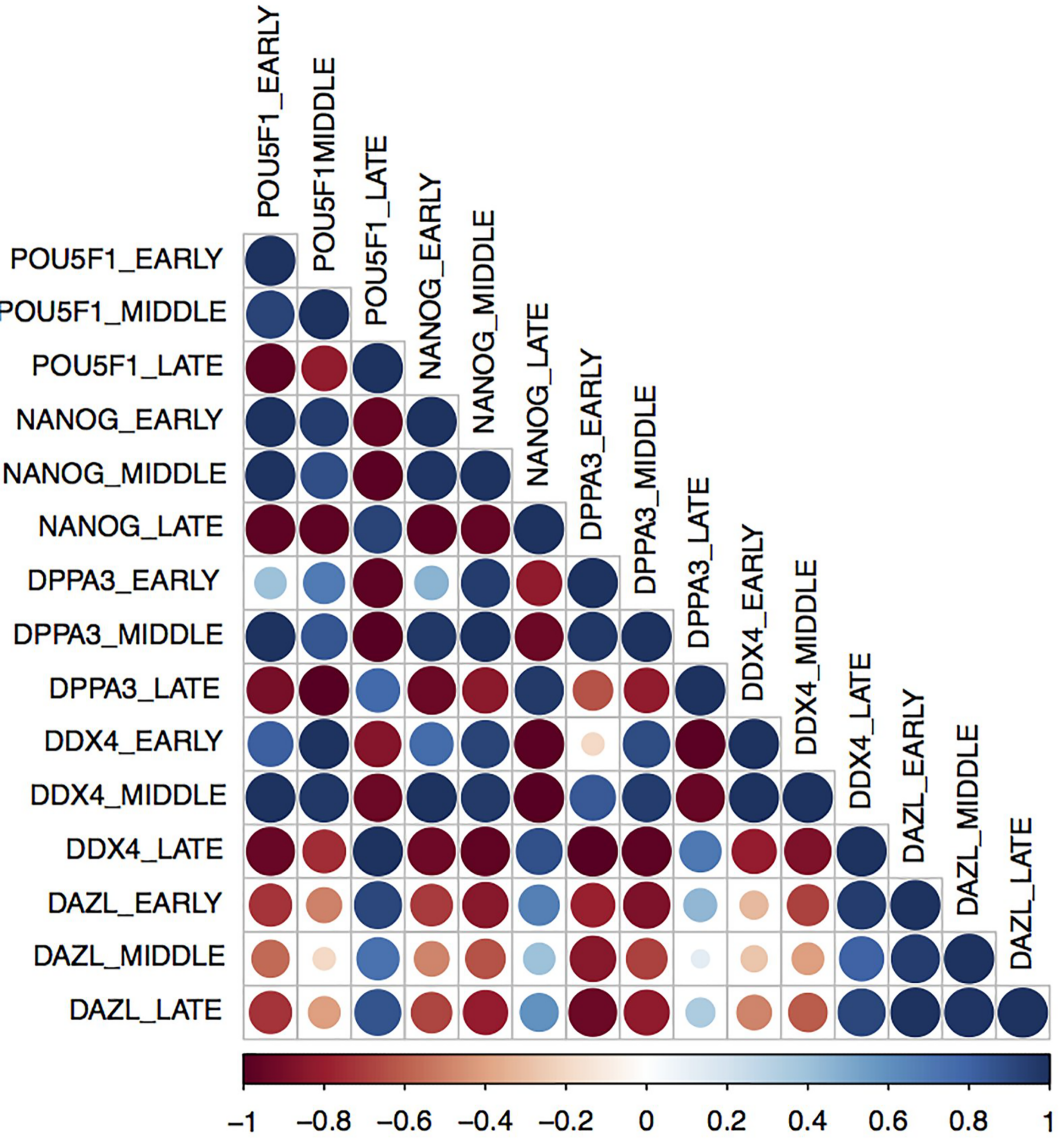
Gene expression correlations determined according to the *Pearson* coefficient. The correlation (*Pearson*) profiles of the pluripotent and germinative genes in the canine PGCs during the early, middle and late periods. Dark blue indicates a positive correlation, and dark red indicates a negative correlation.

The Pearson equation was applied to compare the gene expression in the PGCs during the early, middle, and late periods, and the *POU5F1* gene was correlated (< or > 0.90) to all genes and stages. Furthermore, the expression of *NANOG* was correlated (< or > 0.90) with the expression of *DDPA3* and *DDX4* during all stages. During the early and late periods, the *DDX4* gene was positively associated with only *DAZL*, and the *DPPA3* gene was correlated (< or > 0.90) with the *DDX4* gene (Fig 4) (S4 Table).

### PGCs epigenetic markers during development

**Fig 5:**
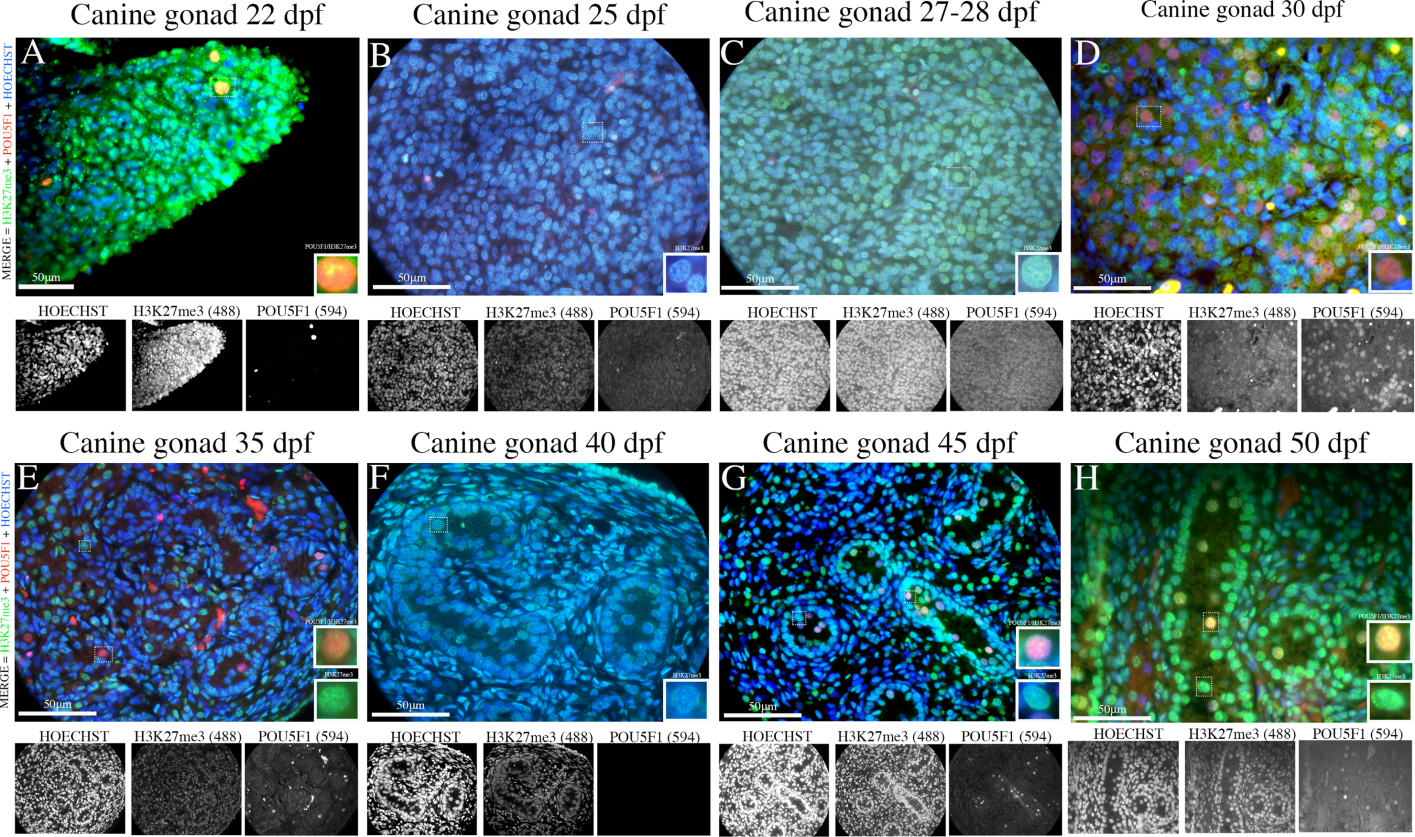
PGCs epigenetic markers during development. (A-H) Sections of the male canine gonadal ridge and PGCs during the early (22–30 dpf), middle (35–40 dpf) and late (45–50 dpf) gestational periods showing the expression of H3K27me3 (green/nuclear) and/or POU5F1 (red/nuclear) and co-localization with POU5F1^+^ (yellow/orange) (Scale bars are 50 μm).

The immunofluorescence epigenetic profiles of the male embryonic and foetal gonads were similar during all periods. H3K27me3^+^ (green) was expressed in the gonadal ridge, gonocytes and foetal testes and co-localized with POU5F1^+^ (yellow/orange) (Fig 5).

**Fig 6:**
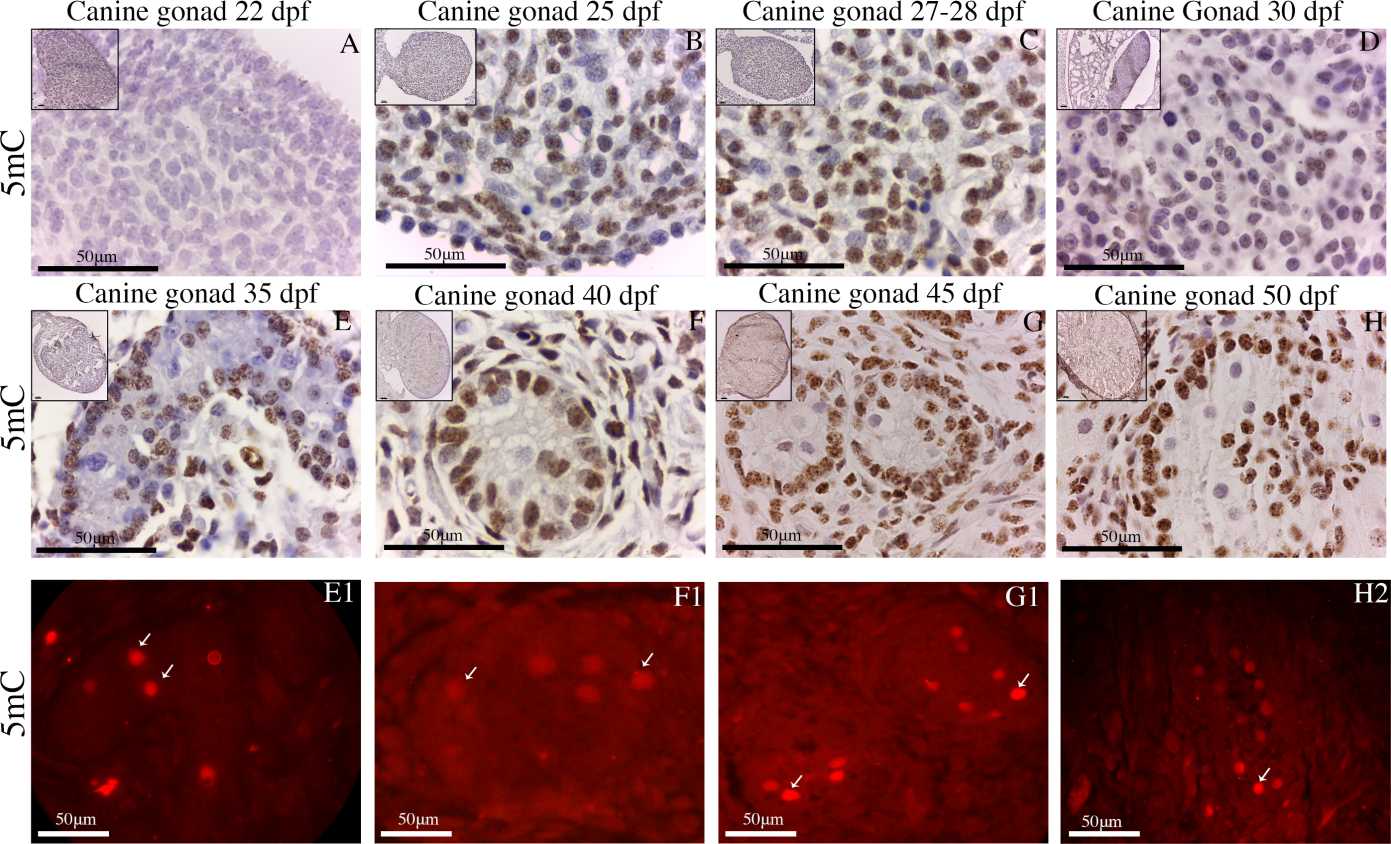
PGCs epigenetic markers during development. (A-H) Sections of the male canine gonadal ridge and PGCs during the early (25–30 dpf), middle (35–40 dpf) and late (45–50 dpf) gestational periods showing the expression of 5mC (dab staining–brown/nuclear). (E-H) 5mC was clearly expressed in the gonocytes and foetal testes even though the spermatogonial cells were not positive for 5mC (white arrow, E to H). (E1-H1) However, the spermatogonial cells continued to show POU5F1 positivity (red/nuclear). (Scale bars are 50 μm).

The 5hmC and H3K9me2 markers were present only in the foetus at 50 days of gestation. 5mC exhibited progressively increasing expression beginning at 22 days of gestation (Fig 6). After this time point, 5mC was clearly expressed in the gonocytes and foetal testes even though the spermatogonial cells were not positive for 5mC (Fig 6). Interestingly, the spermatogonial cells continued to show POU5F1 positivity (red).

### *In vitro* culture and characterization of canine EGCs

**Fig 7:**
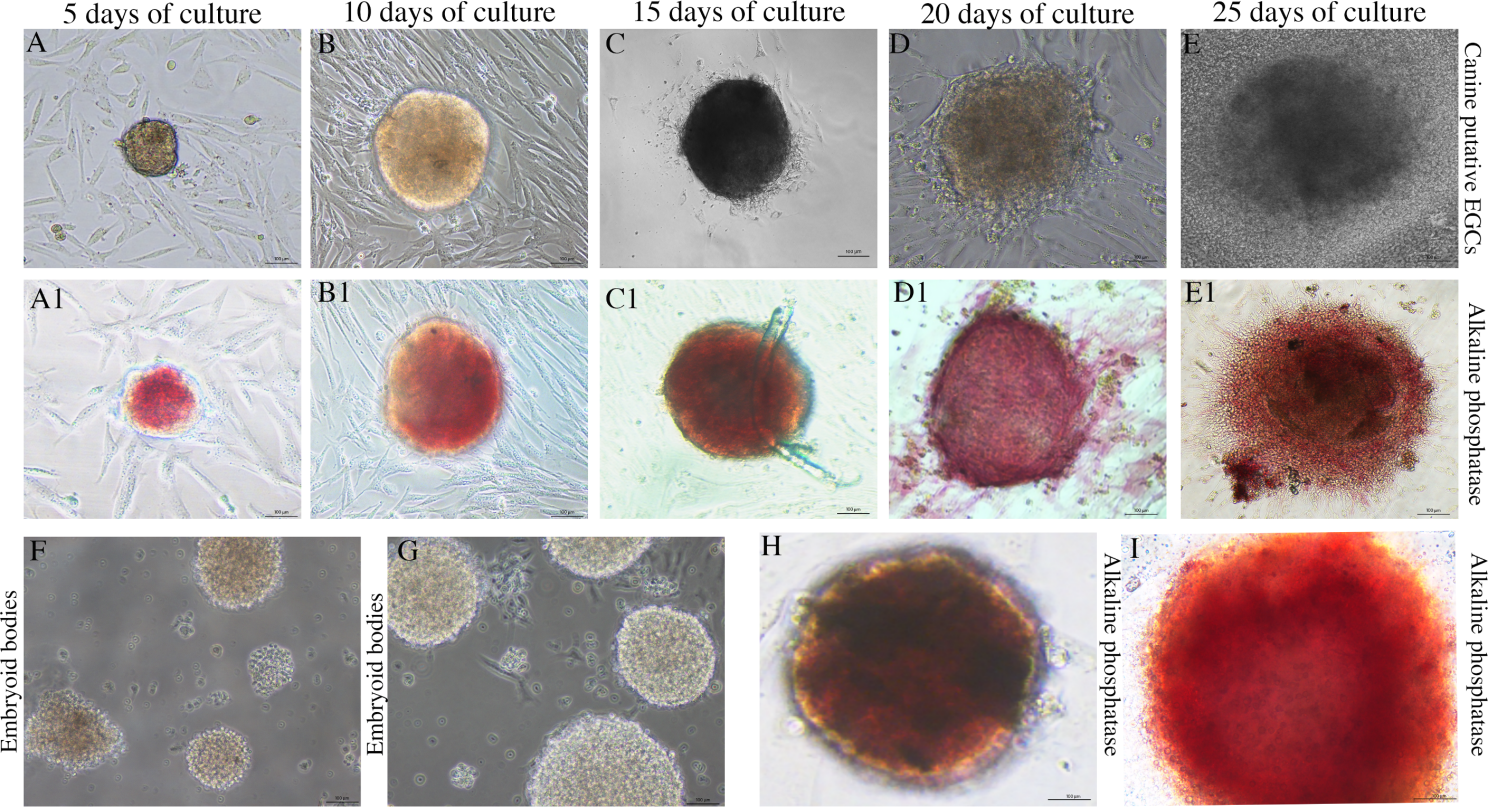
*In vitro* culture of putative canine EGCs. (A-E) Morphology of putative canine EGCs during different periods of culture. (A1- E1) Canine EGCs positively stained for AP. (F-H) EB formation induced by suspension culture of canine EGCs positively stained for AP (Scale bars are 100 μm).

We isolated canine PGCs from the male gonadal ridge after 30 dpf and established a culture of putative canine EGCs. The combination of mechanical dissociation and enzymatic dissociation using EDTA, dispase and hyaluronidase was determined to be the best disaggregation condition that caused no cell injuries. During the cell culture, we observed three different cell characteristics among days 5, 10, 15 and 20 (Fig 7). After five days of culture, the cells exhibited increased growth and began to form colony-like cell aggregates. After 10–15 days, the cells began to form small groups that resembled colonies around the feeder cells. The putative EGCs presented a colony morphology with a round and/or oval flat shape and a well-defined border. After 20 days of culture, the cells continued to exhibit the same morphological characteristics, but no new colonies were detected. After 25 days of culture, the cells exhibited morphological characteristics that were substantially different from those observed in the beginning and had begun to differentiate into other cell types, including fibroblasts and epithelial cells. The supplements LIF, bFGF, and forskolin (in combination) were used to derivate the putative EGCs, and without these supplements, the cells did not form colonies. All putative canine EGCs exhibited strong alkaline phosphatase activity throughout the 20 days of culture; then, their proliferation slowed, and the cells showed weak AP (Fig 7). Putative EGCs not cultured in feeder cells formed suspension colonies with structures similar to those of embryoid bodies that were also positive for AP (Fig 7).

### TEM and SEM analyses

**Fig 8:**
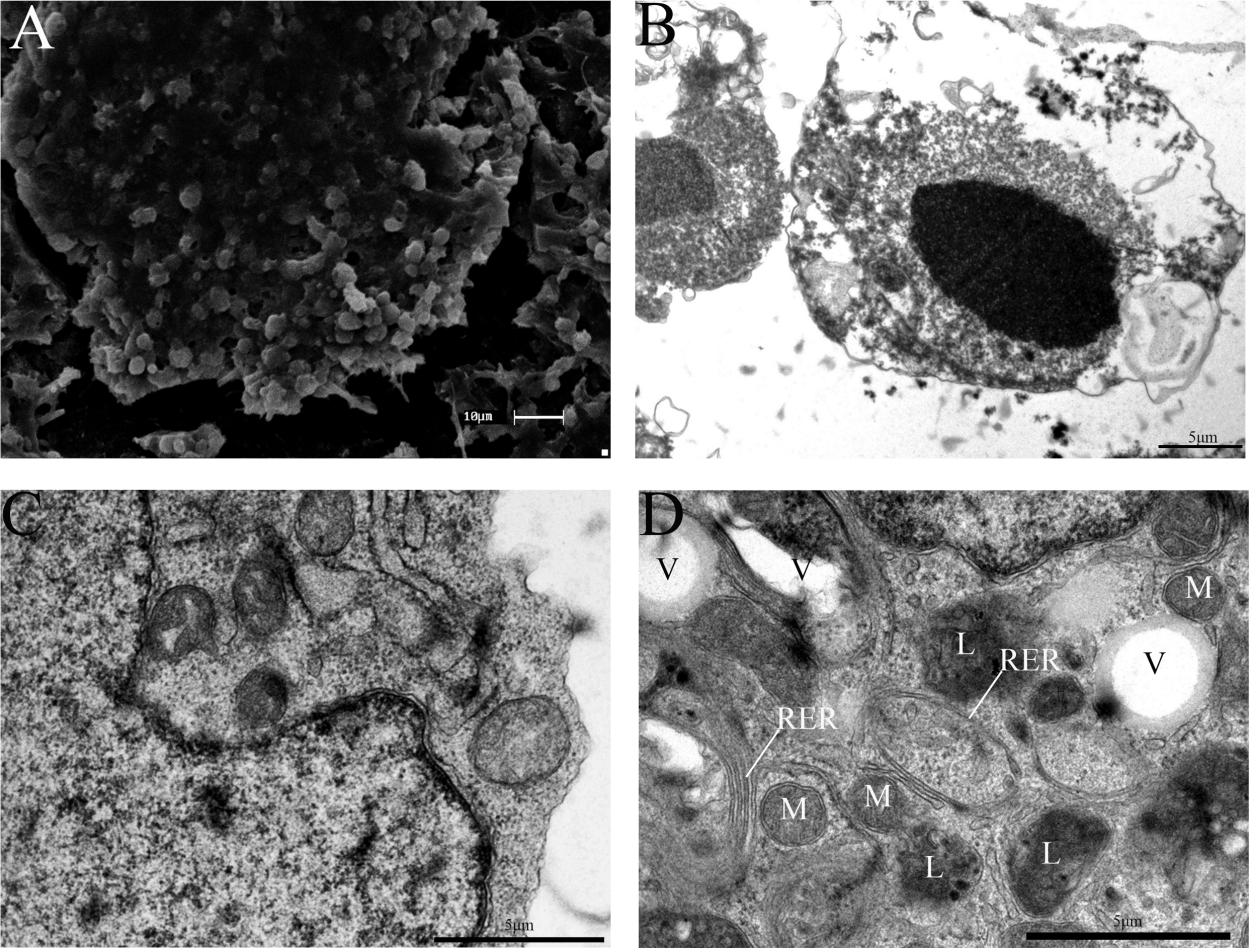
TEM and SEM analyses of canine EGCs. (A) SEM analysis of the peripheral portion of the colony (Scale bar is 100 μm). (B-E) TEM micrograph. (B) Putative EGCs colony with a rounded shape and irregularly shaped cell nuclei. (C and D) Putative EGCs presented many spherical mitochondria (m); vacuoles (v); lysosomes (l) and rough endoplasmic reticulum (rer) (Scale bars are 5 μm).

According to the SEM analysis, the putative EGCs colonies exhibited round and/or oval flat characteristics. The TEM analysis revealed a population of morphologically round cells with a prominent cytoplasm and irregular nucleu. The cells presented abundant mitochondria with spherical and elongated aspects distributed along their cytoplasm. Additionally, the putative EGCs colonies contained endoplasmic reticulum resembling stacked flat vesicles and lysosomes aggregated to the endoplasmic reticulum (Fig 8).

### EGCs immunophenotyping analysis

**Fig 9:**
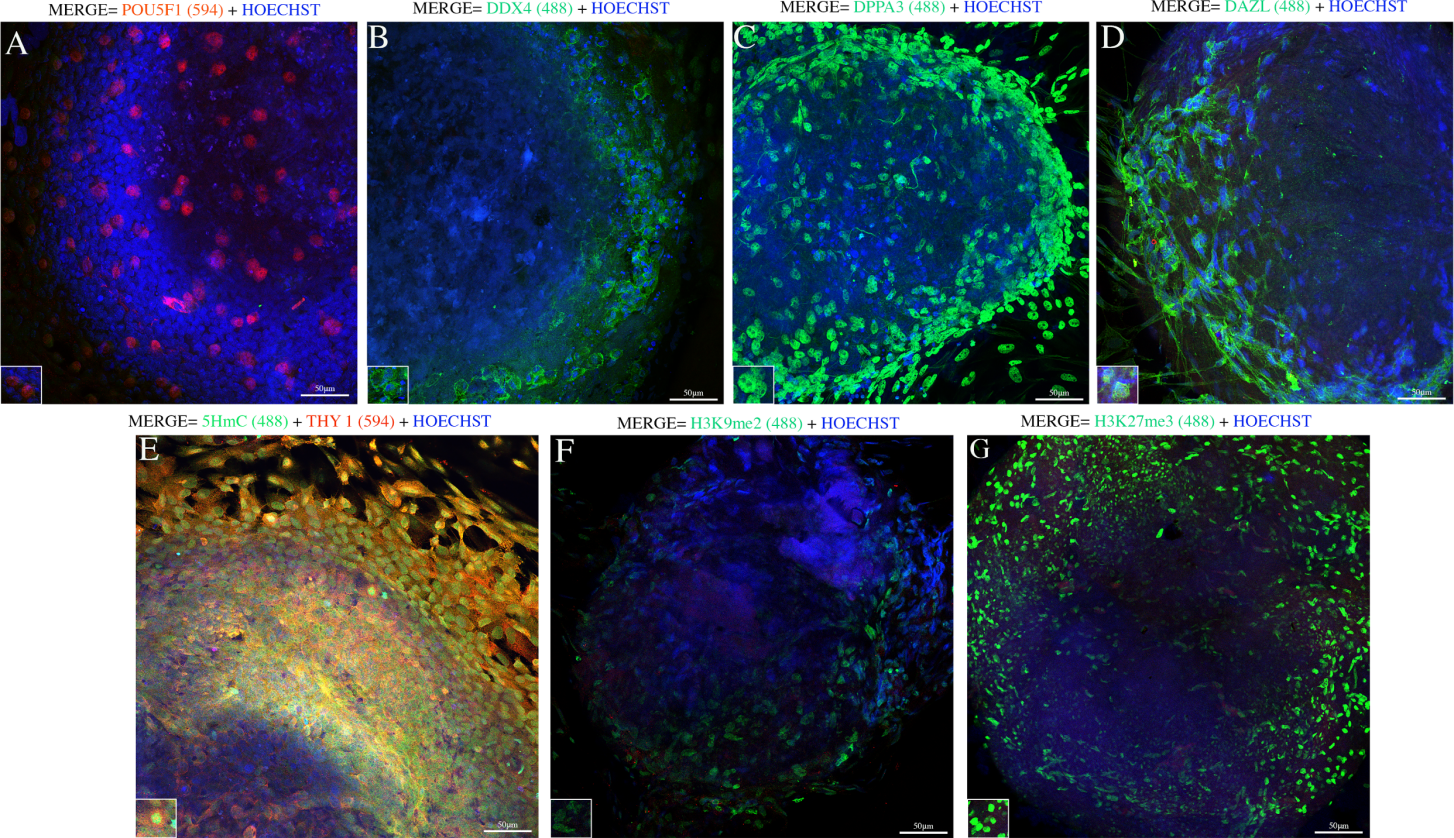
Immunocytochemical analyses of canine EGCs. (A-D) Colony of putative EGCs was identified by the expression of the following: POU5F1 (red/nuclear), DDX4 (green/cytoplasmic), DPPA3 (green/nuclear), and DAZL (green/cytoplasmic). (E-G) EGCs epigenetic markers were identified by the expression of 5hmC (green/ nuclear) and/or THY-1 (red/membrane) (merge of both markers/orange); the canine EGCs were positive for repressive histone H3K27me3 (green/nuclear) and H3K9me2 (green/nuclear). The nuclei were stained with Hoechst (Scale bars are 100 μm).

According to the immunocytochemistry analysis, the cell nuclei of the putative EGCs were positive for the POU5F1 (red) and DPPA3 (green) proteins throughout the cell colony (Fig 9). The cell membranes and cytoplasm were positive for the DDX4 (green) and DAZL (green) proteins (Fig 9). In addition, the THY-1 (red) membrane protein was detected in both the periphery and inside the putative EGCs colonies (Fig 9), suggesting that the colonies consisted mainly of somatic cells, which could explain their rapid differentiation in the culture. The epigenetic profile of the putative EGCs colonies showed intense positivity for the histone marker H3K27me3 (green) and weak positivity for H3K9me2 (green) around the colony periphery (Fig 9). The profile also demonstrated the initiation of DNA demethylation via upregulation of 5-hydroxymethylcytosine (5hmC, Fig 9). 5mC staining was not observed in these cell cultures.

**Fig 10:**
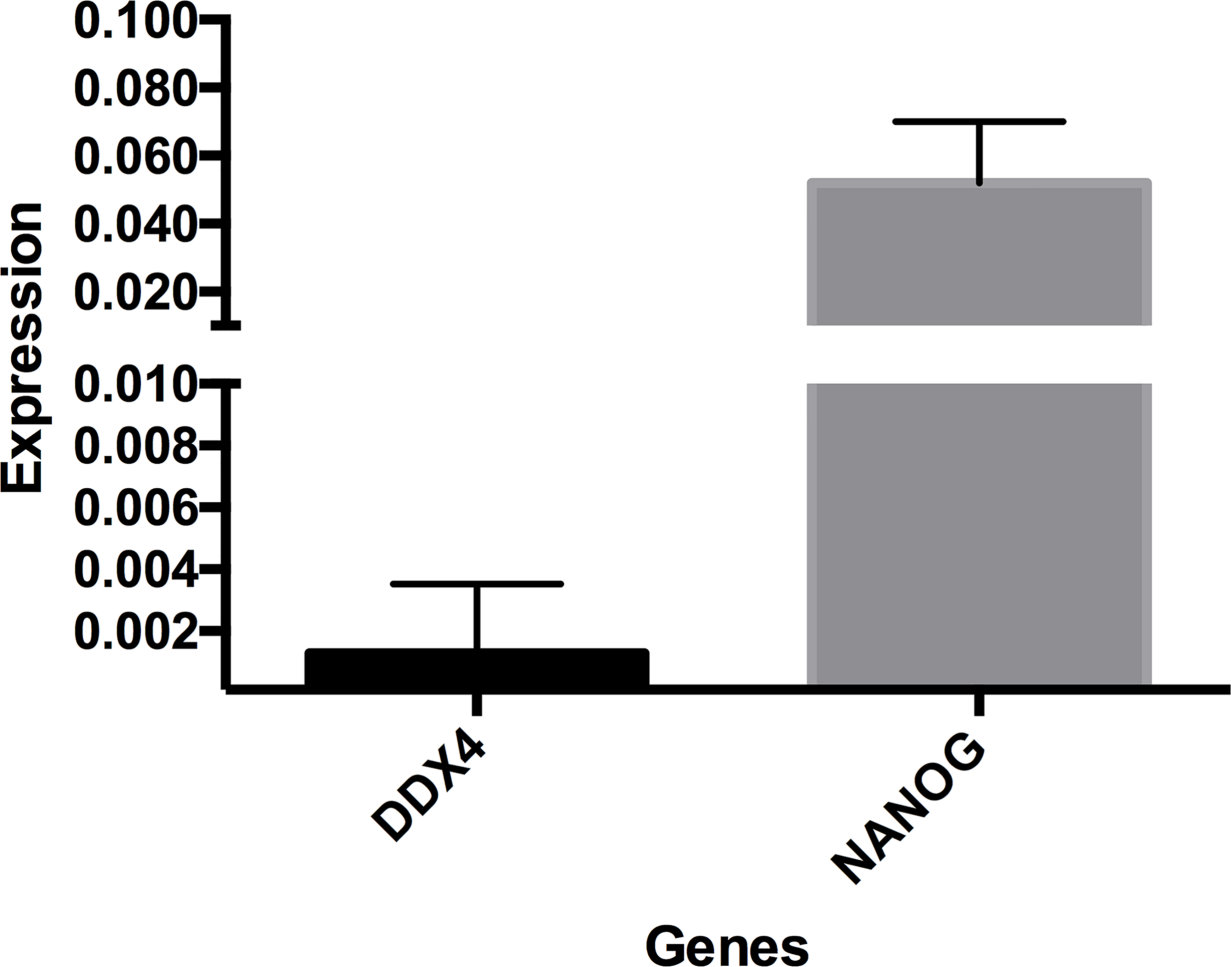
RT-qPCR analyses of pluripotent and germ cell transcripts in putative EGCs. The expression of *NANOG* and *DDX4* was normalized to that of the *GADPH* gene (N = 3 biological samples with technical triplicates for each period, p<0.05).

We measured the transcript levels of the *NANOG* and *DDX4* genes in canine putative EGCs (Fig 10). We were able to detect an abundance of the NANOG gene compared to the *DDX4* gene. The *t-test* analyses revealed significant differences in NANOG (p<0.0490) and DDX4 expression.

## Discussion

### Dynamics of canine PGCs development

Here, we studied and characterized the progression of PGCs during embryonic and foetal development in canines. Specifically, we combined morphological examinations, immunofluorescence characterization, and gene expression analyses to explore the development of cells from the early stage of gonad development to the formation of foetal testes. Importantly, this study demonstrated the ability of pluripotent, germinative and epigenetic markers to identify PGCs *in vivo*. PGCs development has been studies in both mice and humans. However, animal models, such as dogs, require better descriptions, and older data should be updated [33,46]. Additionally, in this study, canine PGCs were detected in the gonadal ridge at 22 days of gestation. Once PGCs enter the gonadal ridge, their morphology gradually changes until the foetal testes are developed. The testes cords prevent meiosis, and male PGCs enter mitotic arrest. Furthermore, these differentiation events are accompanied by changes in the expression of genes, including germ cell-related genes.

Our study revealed the expression of heterogeneous pluripotent, germline and epigenetic markers in PGCs during the three periods of gestation. Positive expression of the pluripotent and germinative marker *POU5F1* has been reported in the gonadal ridge on days 21–25 of canine gestation, which is consistent with our results. Furthermore, we observed that the gene expression levels of *POU5F1* and *NANOG* were significantly increased as the dog PGCs developed. The same pattern has been observed in human PGCs; however, in humans, the *POU5F1* expression detected in seminiferous cord cells was greater during the second trimester of gestation [47]. In contrast, these pluripotency genes were equally homogeneously expressed in mice of both sexes from stage E7.5 to E12.5 [13,48].

During the middle and late periods (35–50 dpf), the specific germ cell marker DAZL was expressed in single germ cells and co-localized with POU5F1. The gene expression of *DAZL* was correlated with that of the *DDX4* gene during the foetal period, suggesting that both markers are potentially required for germ cell maturation and are continuously expressed during the postnatal period in the pre-pubertal testes, which prepares the cells for initiation of the meiotic process [37]. Interestingly, the expression of *DDX4* was undetectable in the male canine PGCs during the early period. A similar result was found in human germ cells during the second trimester of gestation, and the maximum expression of the markers DAZL and DDX4 was observed in male gonads [49]. However, foetal germ cells appear to be a homogenous population during the development of mice testes [8].

DNA methylation reportedly regulates the expression of tissue-specific genes, including in PGCs [1]. In mice, Maatouk showed that the post migratory germ cell-specific genes *MVH* (*DDX4*), *DAZL* and *SCP3* were demethylated in germ cells but not somatic cells [50]. Other mouse studies showed that the *DPPA3* gene is involved in the Tet-mediated active demethylation process during PGCs reprogramming [51]. In contrast, our data show that the *DAZL*, *DDX4*, and *DDPA3* genes can be interconnected to maintain a state of DNA methylation. In particular, our data suggest that DNA demethylation might be related to a gain of POU5F1 because the spermatogonial cells in the canine foetal testes were positive only for POU5F1 and negative for the 5mC DNA methylation marker.

During the period of migration and colonization, the global DNA methylation levels (5mC) decrease in mice [5]. However, the exact moment during which the methylation/demethylation process occurs in canines is not well-described. This study provides the first demonstration that global DNA demethylation occurs in the male canine gonadal ridge during the embryonic stage at 22 days of gestation. Subsequently, the global DNA methylation levels (5mC) increase passively until the foetal testes form. Comparable results have been reported in swine, in which global DNA methylation occurs in the foetal testes [34].

In addition, the expression of the histone marks H3K27me3 and H3K9me2 was detected in the male canine gonads, which has been previously reported [52,53]. As the expression of the histone mark H3K9me2 decreased, the expression of the histone mark H3K27me3 increased, thus enabling the expression of the pluripotent genes [54]. Additionally, we observed that the *NANOG* and *POU5F1* genes were simultaneously expressed as the expression of H3K27me3 increased in the canine PGCs.

### Dynamics of canine EGCs development

The *in vitro* culture and differentiation of germ cells have created excitement among researchers because the establishment of these cell lineages opens new possibilities for transplants, mature gamete production, and other valuable reproduction technologies. Several studies in animals and humans have demonstrated the feasibility of culturing and differentiating germ cells *in vitro* [22,55].

Human EGCs studies have demonstrated that the induction of EGCs from older testes (13-weeks of gestation) is more difficult [37,38]. Thus, we isolated and cultured PGCs at the embryonic developmental stages (30 dpf) to obtain the canine EGCs.

Specific culture conditions are required for EGCs generation because these cells depend on particular supplements, such as LIF, bFGF and forskolin, to increase the number of cells, maintain pluripotency and reduce the levels of apoptosis, allowing cell renewal [39–44, 49–50]. Similarly to humans, mice and swine [19,44,56,57], our results showed that supplementation of the culture conditions with these factors allowed the canine putative EGCs to proliferate and form colonies in cells that were positive for AP. The morphological characteristics of the EGCs colonies were observed by SEM and TEM, and revealed that these colonies consisted of germ cell aggregates with large nuclei, abundant cytoplasm, and numerous mitochondria.

The expression of the pluripotency and germinative markers was characterized in all putative canine EGCs colonies through immunocytochemistry and RT-qPCR assays. The canine EGCs colonies were positive for the markers POU5F1, DDX4, DAZL, DPPA3 and THY-1 and the transcription factor *NANOG*, which is consistent with the previously reported characteristics of the EGCs profile [58,59]. Murakami showed that *NANOG* and cytokines potentially promote the culture of EGCs [60]. Our results suggest that *NANOG* might play a role in PGCs development, which is similar to its role in EGCs development.

The epigenetic profile of the putative canine EGCs showed unique epigenetic modifications, such as the global enrichment of H3K27me3 expression, erasure of H3K9me2 expression, and gain of 5hmC expression. These events are related to the acquisition of the expression of pluripotent genes, such as *NANOG* and *SOX2* [53,61], which is consistent with our results in which the expression of the pluripotent transcription factor *NANOG* was evident in the canine EGCs.

Variations in the EGCs culture have been described mainly in human embryonic germ cells (hEGCs) [62]. In this study, the putative canine EGCs formed colonies that were strongly positive for AP and expressed both pluripotent markers and transcripts, albeit in very low numbers.

## Conclusion

This study provides new and important findings regarding the morphology and expression of pluripotent- and epigenetic-related factors in germ cells during canine embryonic and foetal development. Additionally, the derivation and *in vitro* culture of canine EGCs was possible. We observed heterogeneous germline PGCs and EGCs populations that had specific and development-dependent molecular signatures of the markers POU5F1, NANOG, DDX4, DAZL, and DPPA3. The epigenetic profiles of these cell populations were determined to include the histone repressive markers H3K27me3 and H3K9me2, and changes in the DNA methylation (5mC) and hydroxymethylation of the DNA (5hmC) status were detected. These results not only open new avenues for studying canine reproductive physiology but also serve as models for comparative reproductive biology.

## Supporting information

S1 Fig Morphology and immunofluorescence of the canine embryo at 15 days of gestation (6 mm CR). S1 Fig. (A) Histological section of canine embryo demonstrating the following in the embryonic colon: 1-amnion, 2-neural tube, 3-somito, 4-dorsal aorta and 5-aorta. (B) Canine embryos were identified by the expression of SOX2 (green/nuclear) in the neural tube region. (C) DPPA3 (green/nuclear) positivity in the canine embryo. (D) Epigenetic markers of 5hmC (green/nuclear) were positive in all embryos. (E and F) All embryos were positive for repressive histone H3K27me3 (green/nuclear) and H3K9me2 (green/nuclear). Magnification of 10X (Scale bars are 50 μm).
(TIF)

S2 Fig Immunofluorescence of the canine testes during the pubertal and adult periods. S2 Fig. (A) Pubertal testes (6 months) were identified by the expression of DDX4 (green/cytoplasmic) in Sertoli cells (yellow arrow), spermatogonia (white arrow) and spermatocytes (red arrow). (B) Adult testes (5 years) were identified by the expression of DDX4 (green/cytoplasmic) in differentiated spermatogonial cells (yellow arrow), spermatocytes (red arrow) and rounded spermatids (orange arrow). Magnification of 40X (Scale bars are 50 μm).
(TIF)

S3 Fig Sequence composition and divergence rates. Maximum likelihood tree showing the relationship among the *Canis lupus familiaris*, *Homo sapiens*, *Mus musculus* and *Rattus norvegicus* sequences deposited in GenBank based on the *POU5F1* and *NANOG genes*. The numbers above the nodes indicate the bootstrap confidence levels from the maximum likelihood tree.
(TIF)

S4 Fig Sequence composition and divergence rates. Maximum likelihood tree showing the relationship among the *Canis lupus familiaris*, *Homo sapiens*, *Mus musculus* and *Rattus norvegicus* sequences deposited in GenBank based on the *DAZL*, *DDX4 and DPPA3 genes*. The numbers above the nodes indicate the bootstrap confidence levels from the maximum likelihood tree.
(TIF)

S5 Fig SRY gene electrophoresis. SRY gene electrophoresis of the 271-bp fragment. From right to left, 1 represents the adult canine testis (positive control); 2 represents the adult canine ovary (negative control); 5 represents the male embryo at 15 dpf; 7 represents an embryo at 22 dpf; 8 represents an embryo at 25 dpf; 9 represents an embryo at 27–28 dpf; 10 represents an embryo at 30 dpf; and 2, 3, 4 and 6 represent possible female embryos.
(TIF)

S1 Table **Estimates of evolutionary divergence among *Canis lupus familiaris* (A), *Homo sapiens* (B), *Mus musculus* (C) and *Rattus norvegicus* (D) sequences of *POU5F1*, *NANOG*, *DAZL*, *DDX4* and *DDPA3 genes***. The upper diagonal shows nucleotide differences in relation to the number of bases compared. The lower diagonal shows % pairwise distances identity.
(DOCX)

S2 Table Quantification of canine putative PGCs in the gonadal ridges detected by POU5F1 and DDX4 antibodies. S2 Table. This is the S2 Table Legend ID: Identification; DPF: Days post-fertilization; ST: Section; #PGCs: Total number of PGCs.
(DOC)

S3 Table Quantification of canine PGCs in the gonadal ridges detected by POU5F1 and DAZL antibodies. S3 Table. This is the S3 Table Legend ID: Identification; DPF: Days post-fertilization; ST: Section; #PGCs: Total number of PGC.
(DOC)

S4 Table Quantification of canine PGCs in the gonadal ridges detected by POU5F1 and DDPA3 antibodies. S4 Table. This is the S4 Table Legend ID: Identification; DPF: Days post-fertilization; ST: Section; #PGCs: Total number of PGC.
(DOC)

S5 Table Correlational (*Pearson*) profiles of pluripotent and germinative genes in canine PGCs during the early, middle and late periods. (DOCX)
