# Supplementary figures and images for: Dynamics of male canine germ cell development
Source: PLoS One. 2018 Feb 28;13(2):e0193026. doi: 10.1371/journal.pone.0193026 (PMC5831030; doi:10.1371/journal.pone.0193026)

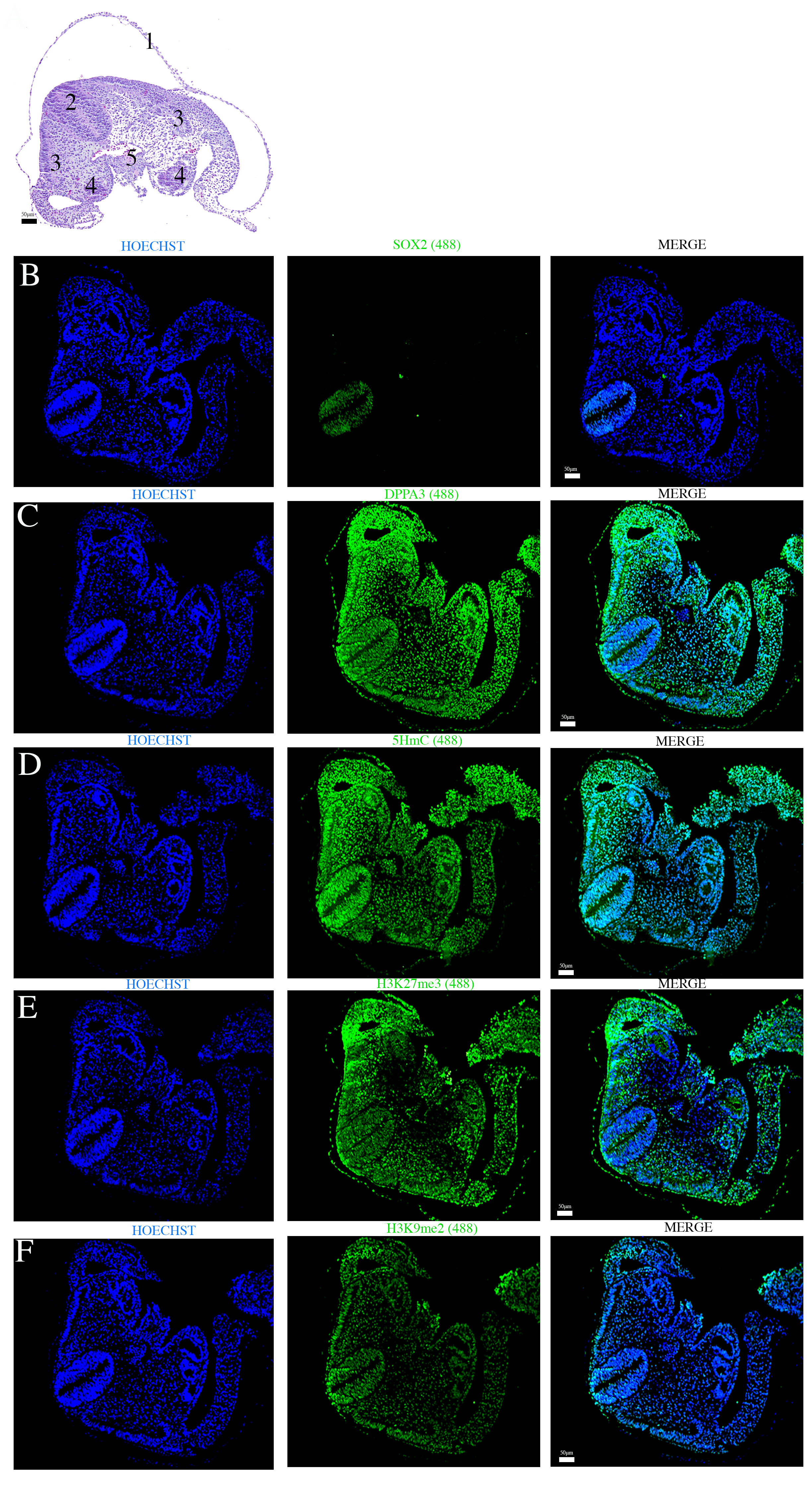

Supplement: S1 Fig — (A) Histological section of canine embryo demonstrating the following in the embryonic colon: 1-amnion, 2-neural tube, 3-somito, 4-dorsal aorta and 5-aorta. (B) Canine embryos were identified by the expression of SOX2 (green/nuclear) in the neural tube region. (C) DPPA3 (green/nuclear) positivity in the canine embryo. (D) Epigenetic markers of 5hmC (green/nuclear) were positive in all embryos. (E and F) All embryos were positive for repressive histone H3K27me3 (green/nuclear) and H3K9me2 (green/nuclear). Magnification of 10X (Scale bars are 50 μm). (TIF) [file pone.0193026.s001.tif]

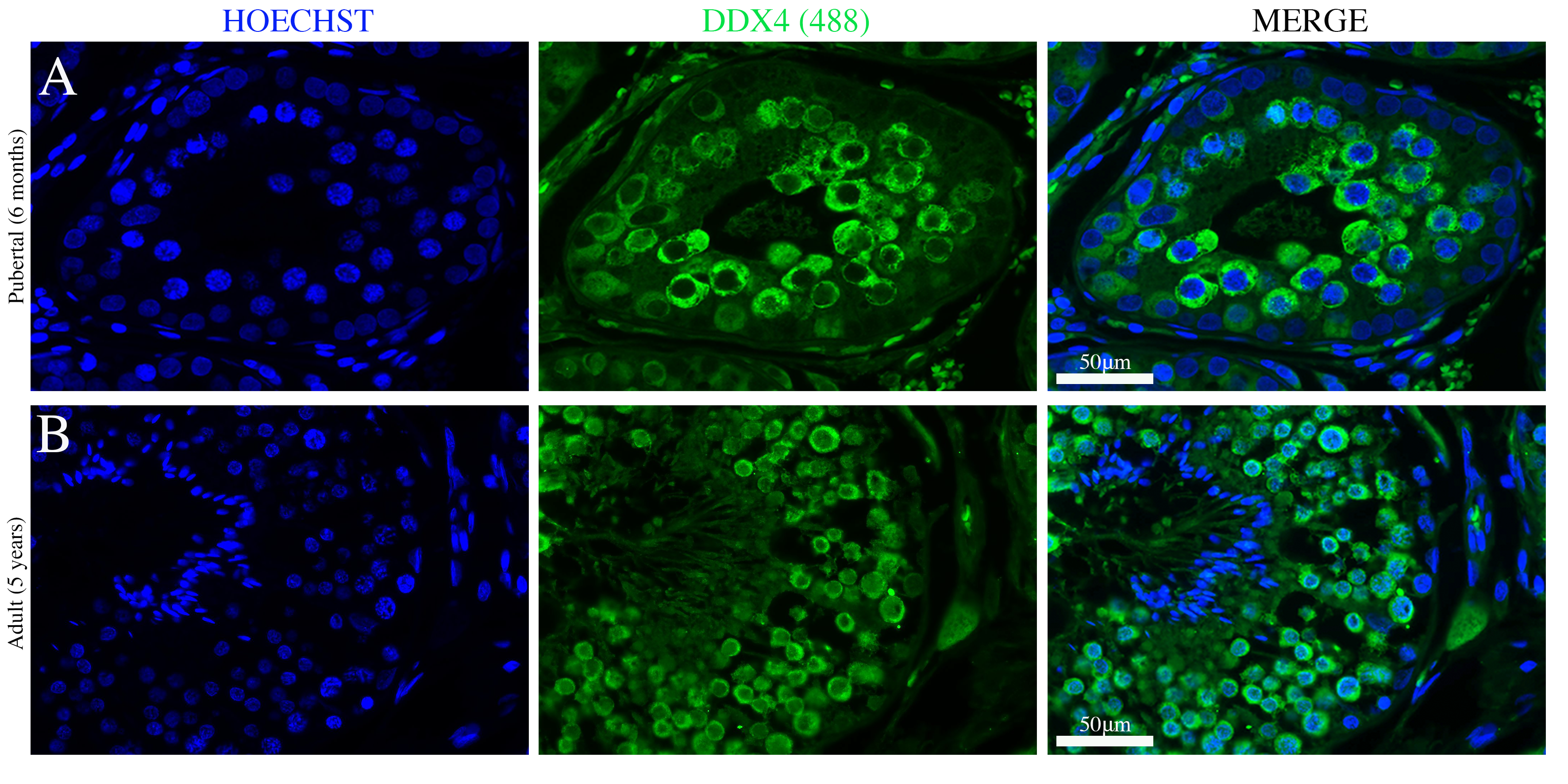

Supplement: S2 Fig — (A) Pubertal testes (6 months) were identified by the expression of DDX4 (green/cytoplasmic) in Sertoli cells (yellow arrow), spermatogonia (white arrow) and spermatocytes (red arrow). (B) Adult testes (5 years) were identified by the expression of DDX4 (green/cytoplasmic) in differentiated spermatogonial cells (yellow arrow), spermatocytes (red arrow) and rounded spermatids (orange arrow). Magnification of 40X (Scale bars are 50 μm). (TIF) [file pone.0193026.s002.tif]

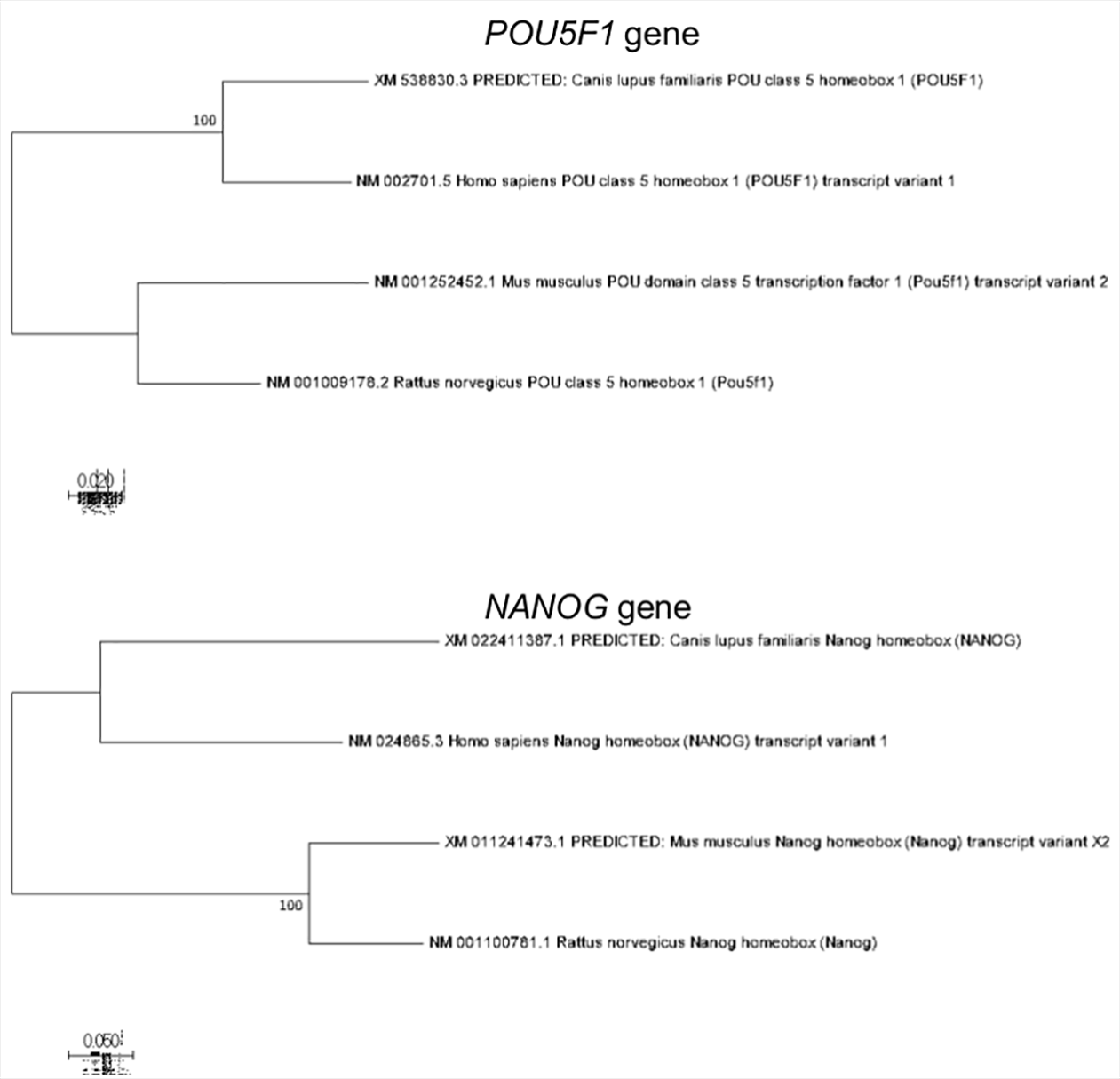

Supplement: S3 Fig — Maximum likelihood tree showing the relationship among the Canis lupus familiaris, Homo sapiens, Mus musculus and Rattus norvegicus sequences deposited in GenBank based on the POU5F1 and NANOG genes. The numbers above the nodes indicate the bootstrap confidence levels from the maximum likelihood tree. (TIF) [file pone.0193026.s003.tif]

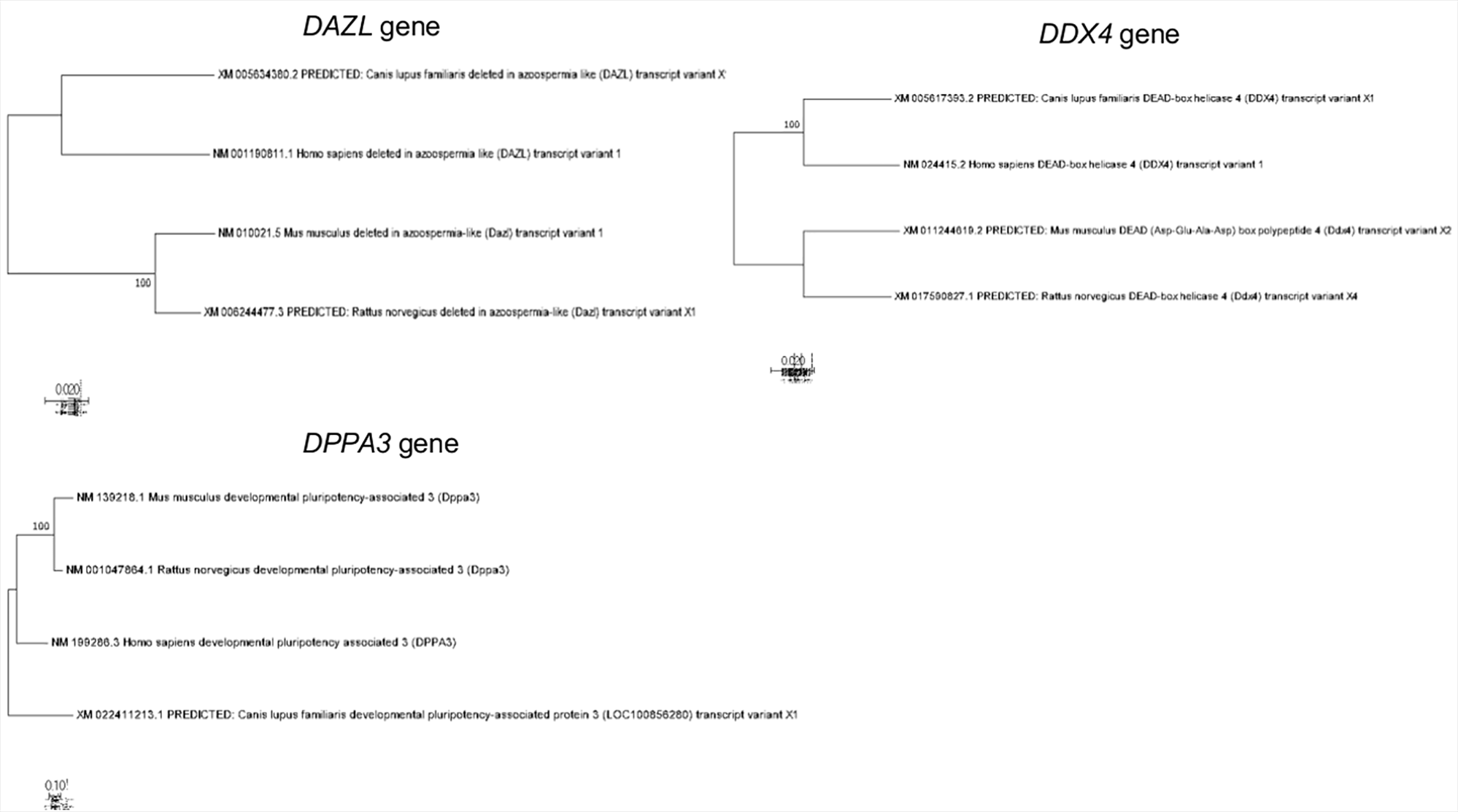

Supplement: S4 Fig — Maximum likelihood tree showing the relationship among the Canis lupus familiaris, Homo sapiens, Mus musculus and Rattus norvegicus sequences deposited in GenBank based on the DAZL, DDX4 and DPPA3 genes. The numbers above the nodes indicate the bootstrap confidence levels from the maximum likelihood tree. (TIF) [file pone.0193026.s004.tif]

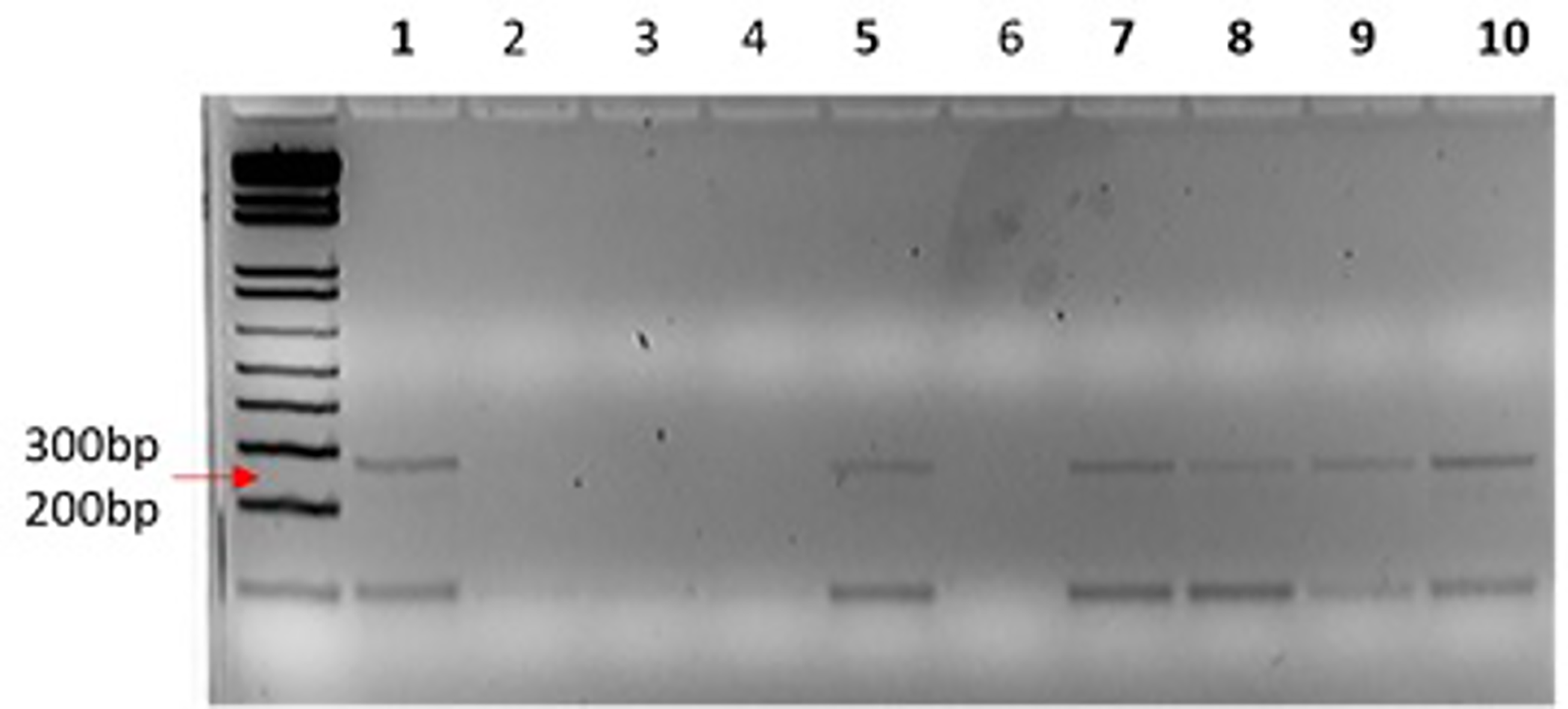

Supplement: S5 Fig — SRY gene electrophoresis of the 271-bp fragment. From right to left, 1 represents the adult canine testis (positive control); 2 represents the adult canine ovary (negative control); 5 represents the male embryo at 15 dpf; 7 represents an embryo at 22 dpf; 8 represents an embryo at 25 dpf; 9 represents an embryo at 27–28 dpf; 10 represents an embryo at 30 dpf; and 2, 3, 4 and 6 represent possible female embryos. (TIF) [file pone.0193026.s005.tif]
