# Supplementary material for: Dynamics of male canine germ cell development
Source: PLoS One. 2018 Feb 28;13(2):e0193026. doi: 10.1371/journal.pone.0193026 (PMC5831030; doi:10.1371/journal.pone.0193026)
Supplement: S1 Table — Estimates of evolutionary divergence among Canis lupus familiaris (A), Homo sapiens (B), Mus musculus (C) and Rattus norvegicus (D) sequences of POU5F1, NANOG, DAZL, DDX4 and DDPA3 genes. The upper diagonal shows nucleotide differences in relation to the number of bases compared. The lower diagonal shows % pairwise distances identity. (DOCX) [file pone.0193026.s006.docx]

**S1 Table**. Estimates of evolutionary divergence among *Canis lupus familiaris* (A), *Homo sapiens* (B), *Mus musculus* (C) and *Rattus norvegicus* (D*)* sequences of *POU5F1, NANOG, DAZL, DDX4* and *DDPA3 genes*. The area above the diagonal shows nucleotide differences in relation to the number of bases compared. The area below the diagonal shows % pairwise distance identity.

| **Genes** | **Species** | **A** | **B** | **C** | **D** |
| --- | --- | --- | --- | --- | --- |
| *POU5F1* | A | - | 93 | 215 | 188 |
|  | B | 9.6 | - | 212 | 182 |
|  | C | 22.2 | 21.9 | - | 117 |
|  | D | 19.4 | 18.8 | 12.1 | - |
| *NANOG* | A | - | 382 | 500 | 494 |
|  | B | 33.2 | - | 460 | 454 |
|  | C | 43.5 | 40.0 | - | 191 |
|  | D | 43.0 | 39.5 | 16.6 | - |
| *DAZL* | A | - | 262 | 349 | 336 |
|  | B | 12.6 | - | 342 | 335 |
|  | C | 16.8 | 16.5 | - | 98 |
|  | D | 16.2 | 16.1 | 4.7 | - |
| *DDX4* | A | - | 177 | 294 | 291 |
|  | B | 8.0 | - | 304 | 295 |
|  | C | 13.4 | 13.8 | - | 177 |
|  | D | 13.2 | 13.4 | 4.7 | - |
| *DPPA3* | A | - | 334 | 373 | 354 |
|  | B | 51.1 | - | 300 | 287 |
|  | C | 57.1 | 45.9 | - | 131 |
|  | D | 54.2 | 44.0 | 20.1 | - |
