# Supplementary material for: Dynamics of male canine germ cell development
Source: PLoS One. 2018 Feb 28;13(2):e0193026. doi: 10.1371/journal.pone.0193026 (PMC5831030; doi:10.1371/journal.pone.0193026)
Supplement: S2 Table — This is the S2 Table Legend ID: Identification; DPF: Days post-fertilization; ST: Section; #PGCs: Total number of PGCs. (DOC) [file pone.0193026.s007.doc]

**S2 Table**. Quantification of canine putative PGCs in the gonadal ridges detected by POU5F1 and DDX4 antibodies.

| **ID** | **Size** | **DPF** | **ST** | **POU5F1 +** | **%** | **DDX4+** | **%** | **POU5F1+ DDX4+** | **%** | **#PGCs** |
| --- | --- | --- | --- | --- | --- | --- | --- | --- | --- | --- |
| AQ | 1 | 22 | 3 | 19 | 100 | 0 | 0 | 0 | 0 | 19 |
| AB | 1.5 | 25-26 | 3 | 38 | 100 | 0 | 0 | 0 | 0 | 38 |
| AF | 1.5 | 25-26 | 3 | 42 | 100 | 0 | 0 | 0 | 0 | 42 |
| AL | 1.5 | 25-26 | 3 | 35 | 100 | 0 | 0 | 0 | 0 | 35 |
| AC | 2 | 27-28 | 3 | 49 | 96.07 | 1 | 3.92 | 0 | 0 | 50 |
| AD | 3 | 30 | 3 | 196 | 92.89 | 3 | 1.50 | 0 | 0 | 199 |
| AE | 3 | 30 | 3 | 210 | 91.70 | 2 | 8.29 | 0 | 0 | 212 |
| AO | 3.5 | 35 | 3 | 92 | 86.69 | 8 | 8 | 0 | 0 | 100 |
| BA | 4 | 40 | 3 | 43 | 82.69 | 9 | 17.30 | 0 | 0 | 52 |
| BA-1 | 4 | 40 | 3 | 37 | 84.09 | 7 | 15.90 | 0 | 0 | 44 |
| AE-1 | 7 | 45 | 3 | 38 | 74.50 | 13 | 25.49 | 0 | 0 | 51 |
| AE-3 | 7 | 45 | 3 | 40 | 80 | 10 | 20 | 0 | 0 | 50 |
| AZ | 9.5 | 50 | 3 | 43 | 56.57 | 24 | 31.57 | 9 | 11.84 | 76 |
| AZ-1 | 9.5 | 50 | 3 | 45 | 61.64 | 21 | 28.76 | 7 | 9.58 | 73 |

ID: Identification; DPF: Days post-fertilization; ST: Section; #PGCs: Total number of PGCs.
