# Supplementary material for: Dynamics of male canine germ cell development
Source: PLoS One. 2018 Feb 28;13(2):e0193026. doi: 10.1371/journal.pone.0193026 (PMC5831030; doi:10.1371/journal.pone.0193026)
Supplement: S3 Table — This is the S3 Table Legend ID: Identification; DPF: Days post-fertilization; ST: Section; #PGCs: Total number of PGC. (DOC) [file pone.0193026.s008.doc]

**S3 Table**. Quantification of canine PGCs in the gonadal ridges detected by POU5F1 and DAZL antibodies.

| **ID** | **Size** | **DPF** | **ST** | **POU5F1 +** | **%** | **DAZL+** | **%** | **POU5F1+ DAZL+** | **%** | **#PGCs** |
| --- | --- | --- | --- | --- | --- | --- | --- | --- | --- | --- |
| AQ | 1 | 22 | 3 | 19 | 100 | 0 | 0 | 0 | 0 | 19 |
| AB | 1.5 | 25-26 | 3 | 38 | 100 | 0 | 0 | 0 | 0 | 38 |
| AF | 1.5 | 25-26 | 3 | 42 | 100 | 0 | 0 | 0 | 0 | 42 |
| AL | 1.5 | 25-26 | 3 | 35 | 100 | 0 | 0 | 0 | 0 | 35 |
| AC | 2 | 27-28 | 3 | 49 | 96.07 | 1 | 3.92 | 0 | 0 | 50 |
| AD | 3 | 30 | 3 | 200 | 43.48 | 180 | 39.13 | 80 | 17.39 | 460 |
| AE | 3 | 30 | 3 | 193 | 43.46 | 174 | 39.19 | 77 | 17.3 | 444 |
| AO | 3.5 | 35 | 3 | 87 | 36.70 | 150 | 63.29 | 0 | 0 | 237 |
| BA | 4 | 40 | 3 | 39 | 17.41 | 185 | 82.59 | 0 | 0 | 224 |
| BA-1 | 4 | 40 | 3 | 37 | 16.22 | 191 | 83.77 | 0 | 0 | 228 |
| AE-1 | 7 | 45 | 3 | 40 | 16.45 | 193 | 83.55 | 0 | 0 | 231 |
| AE-3 | 7 | 45 | 3 | 40 | 17.54 | 188 | 82.46 | 0 | 0 | 228 |
| AZ | 9.5 | 50 | 3 | 47 | 15.71 | 216 | 72.24 | 36 | 12 | 299 |
| AZ-1 | 9.5 | 50 | 3 | 44 | 15.82 | 205 | 73.74 | 29 | 10.4 | 278 |

ID: Identification; DG: DPF: Days post-fertilization; ST: Section; #PGCs: Total number of PGCs.
