# Supplementary material for: Dynamics of male canine germ cell development
Source: PLoS One. 2018 Feb 28;13(2):e0193026. doi: 10.1371/journal.pone.0193026 (PMC5831030; doi:10.1371/journal.pone.0193026)
Supplement: S4 Table — This is the S4 Table Legend ID: Identification; DPF: Days post-fertilization; ST: Section; #PGCs: Total number of PGC. (DOC) [file pone.0193026.s009.doc]

**S4 Table**. Quantification of canine PGCs in the gonadal ridges detected by POU5F1 and DDPA3 antibodies.

| **ID** | **Size** | **DPF** | **ST** | **POU5F1 +** | **%** | **DPPA3+** | **%** | **POU5F1+ DPPA3+** | **%** | **#PGCs** |
| --- | --- | --- | --- | --- | --- | --- | --- | --- | --- | --- |
| AQ | 1 | 22 | 3 | 17 | 100 | 0 | 0 | 0 | 0 | 17 |
| AB | 1.5 | 25-26 | 3 | 33 | 100 | 0 | 0 | 0 | 0 | 33 |
| AF | 1.5 | 25-26 | 3 | 39 | 100 | 0 | 0 | 0 | 0 | 39 |
| AL | 1.5 | 25-26 | 3 | 33 | 100 | 0 | 0 | 0 | 0 | 33 |
| AC | 2 | 27-28 | 3 | 41 | 100 | 0 | 0 | 0 | 0 | 41 |
| AD | 3 | 30 | 3 | 197 | 100 | 0 | 0 | 0 | 0 | 197 |
| AE | 3 | 30 | 3 | 194 | 100 | 0 | 0 | 0 | 0 | 194 |
| AO | 3.5 | 35 | 3 | 79 | 100 | 0 | 0 | 0 | 0 | 79 |
| BA | 4 | 40 | 3 | 37 | 100 | 0 | 0 | 0 | 0 | 37 |
| BA-1 | 4 | 40 | 3 | 41 | 100 | 0 | 0 | 0 | 0 | 41 |
| AE-1 | 7 | 45 | 3 | 39 | 0.55 | 6.949 | 99.44 | 0 | 0 | 231 |
| AE-3 | 7 | 45 | 3 | 43 | 0.61 | 6.954 | 99.38 | 0 | 0 | 228 |
| AZ | 9.5 | 50 | 3 | 41 | 0.58 | 6.956 | 99.28 | 9 | 0.12 | 7.006 |
| AZ-1 | 9.5 | 50 | 3 | 42 | 0.60 | 6.943 | 99.29 | 7 | 0.10 | 6.992 |

ID: Identification; DG: DPF: Days post-fertilization; ST: Section; #PGCs: Total number of PGCs.
