# Supplementary material for: Dynamics of male canine germ cell development
Source: PLoS One. 2018 Feb 28;13(2):e0193026. doi: 10.1371/journal.pone.0193026 (PMC5831030; doi:10.1371/journal.pone.0193026)
Supplement: S5 Table — (DOCX) [file pone.0193026.s010.docx]

**S5 Table**. The correlation (*Pearson*) profiles of pluripotent and germinative genes of canine PGCs in early, middle and late periods.

| **GENES/PERIODS** | **POU5F1 EARLY** | **POU5F1MIDLE** | **POU5F1 LATE** | **NANOG EARLY** | **NANOG MIDLE** | **NANOG LATE** | **DPPA3 EARLY** | **DPPA3 MIDLE** | **DPPA3 LATE** | **DDX4 EARLY** | **DDX4 MIDLE** | **DDX4 LATE** | **DAZL EARLY** | **DAZL MIDLE** | **DAZL LATE** |
| --- | --- | --- | --- | --- | --- | --- | --- | --- | --- | --- | --- | --- | --- | --- | --- |
| POU5F1 EARLY | 1.00 |  |  |  |  |  |  |  |  |  |  |  |  |  |  |
| POU5F1MIDLE | 0.92 | 1.00 |  |  |  |  |  |  |  |  |  |  |  |  |  |
| POU5F1 LATE | -0.98 | -0.82 | 1.00 |  |  |  |  |  |  |  |  |  |  |  |  |
| NANOG EARLY | 0.99 | 0.95 | -0.96 | 1.00 |  |  |  |  |  |  |  |  |  |  |  |
| NANOG MIDLE | 1.00 | 0.88 | -0.99 | 0.98 | 1.00 |  |  |  |  |  |  |  |  |  |  |
| NANOG LATE | -0.98 | -0.98 | 0.92 | -0.99 | -0.96 | 1.00 |  |  |  |  |  |  |  |  |  |
| DPPA3 EARLY | 0.40 | 0.69 | -0.98 | 0.46 | 0.95 | -0.83 | 1.00 |  |  |  |  |  |  |  |  |
| DPPA3 MIDLE | 0.99 | 0.85 | -1.00 | 0.97 | 1.00 | -0.94 | 0.97 | 1.00 |  |  |  |  |  |  |  |
| DPPA3 LATE | -0.89 | -1.00 | 0.77 | -0.93 | -0.84 | 0.96 | -0.64 | -0.81 | 1.00 |  |  |  |  |  |  |
| DDX4 EARLY | 0.82 | 1.00 | -0.86 | 0.76 | 0.92 | -0.99 | -0.20 | 0.89 | -0.99 | 1.00 |  |  |  |  |  |
| DDX4 MIDLE | 0.99 | 0.97 | -0.93 | 1.00 | 0.96 | -1.00 | 0.84 | 0.95 | -0.95 | 0.99 | 1.00 |  |  |  |  |
| DDX4 LATE | -0.95 | -0.75 | 0.99 | -0.92 | -0.97 | 0.87 | -1.00 | -0.98 | 0.70 | -0.80 | -0.88 | 1.00 |  |  |  |
| DAZL EARLY | -0.73 | -0.50 | 0.91 | -0.71 | -0.85 | 0.67 | -0.78 | -0.88 | 0.44 | -0.32 | -0.69 | 0.95 | 1.00 |  |  |
| DAZL MIDLE | -0.57 | -0.20 | 0.73 | -0.49 | -0.64 | 0.40 | -0.85 | -0.68 | 0.13 | -0.28 | -0.41 | 0.80 | 0.95 | 1.00 |  |
| DAZL LATE | -0.73 | -0.41 | 0.86 | -0.67 | -0.80 | 0.59 | -0.94 | -0.83 | 0.34 | -0.49 | -0.61 | 0.91 | 0.99 | 0.98 | 1.00 |
